# Synthetic Integration of an FCS into Coronaviruses—Hype or an Unresolved Biorisk? An Integrative Analysis of DNA Repair, Cancer Research, Drug Development, and Escape Mutant Traits

**DOI:** 10.3390/life16020199

**Published:** 2026-01-25

**Authors:** Siguna Mueller

**Affiliations:** Center for Research in Medical Pharmacology, University of Insubria, 21100 Varese, Italy; smuellermueller@uninsubria.it

**Keywords:** CoV recombination, RNA virus, viral nuclear role, DNA repair, viruses in cancer research, escape mutants, siRNAs, automation, biorisk policies

## Abstract

A 19 nt fragment that spans the SARS-CoV-2 furin cleavage site (FCS) is identical to the reverse complement of a proprietary human DNA repair gene sequence. Rather than interpreting this overlap as evidence of a laboratory event, this article uses it as a theoretical springboard to explore underappreciated biorisk concerns, specifically in the context of cancer research. Although they are RNA viruses, coronaviruses are capable of hijacking host DNA damage response (DDR) pathways, exploiting nuclear functions to enhance replication and evade innate immunity. Under selective pressures (antivirals, DDR antagonists, or large-scale siRNA libraries designed to silence critical host genes), escape mutants may arise with fitness advantages. Parallel observations involving in vivo RNA interference via chimeric viruses lend plausibility to some of the key aspects underlying unappreciated biorisks. The mechanistic insights that incorporate DNA repair mechanisms, CoVs in the nucleus, specifics of viruses in cancer research, anticancer drugs, automated gene silencing experiments, and gene sequence overlaps identify gaps in biorisk policies, even those unaccounted for by the potent “Sequences of Concern” paradigm. Key concerning attributes, including genome multifunctionality, such as NLS/FCS in SARS-CoV-2, antisense sequences, and their combination, are further described in more general terms. The article concludes with recommendations pairing modern technical safeguards with enduring ethical principles.

## 1. Motivation

A famous passage in the Old Testament recounts the story of King Solomon and two women who both claimed to be the mother of a living infant. The two women had given birth within a few days of each other and lived in the same house, with no other adults present. One woman’s child had died, and in the night, she took the other woman’s living child, placing the dead child in her own bed. The next morning, the mother of the living child discovered the switch. One cannot imagine the dispute that emerged. When the case was presented before King Solomon, he commanded a sword to be brought and ordered the living child to be divided in two, so each woman could have half. What unfolded next has informed numerous realms of justice, psychology, leadership, and ethics (1 Kings 3:16-28).

Then the woman whose son was alive said to the king, because her heart yearned for her son, “Oh, my lord, give her the living child, and by no means slay it.” But the other said, “It shall be neither mine nor yours; divide it.” The king responded, “Give the living baby to the first woman, and don’t kill him. She is his mother.”

This article highlights how technological advances have placed us at a critical juncture. Success in synthetic biology inadvertently results in an “indistinguishability” of opposing attributes, where identifying key agents or even motivations becomes increasingly difficult. Automation and synthetic biology have simplified the creation of biological weapons, largely removing the need for an exuberant laboratory setting and expertise. It has also become easier to camouflage malicious activities, agents, and divert intelligence for nefarious purposes. Various scenarios may not easily be recognized for their dual-use potential.

A curious situation has arisen related to SARS-CoV-2. Specifically, the hypothesis that a 19 ntd genome portion that encompasses the furin cleavage site (FCS) of SARS-CoV-2 is the result of a laboratory recombination [1] is often interpreted to imply a link to the laboratory genesis of this virus.

The sequence is 100% identical to a reverse-complement sequence described in the sequence listing (SEQ ID11652, https://www.ncbi.nlm.nih.gov/nuccore/KH664781.1, last accessed on 17 January 2026) related to a Moderna patent filed on 4 February 2016 [2]. SEQ ID11652 is transcribed into the human mutS homolog (MSH3), which is associated with a critical component of the human DNA mismatch repair (MMR) pathway.

Nonetheless, there are no reasonable indicators that SARS-CoV-2 was deliberately engineered as a bioweapon. Hypothetically, the sequence insert may have unfolded unintentionally during some lab experiments [1]. Still, this sequence does not prove the genesis of SARS-CoV-2. The FCS, despite its importance, is only one part of the virus. Even if this part appears synthetically generated, one may argue about the identity and origin of the viral backbone. Some may wonder whether the genetic sequence, once it had resulted in a recombination event in some CoV, was further used and potentially inserted into a SARS-CoV-2 precursor via synthetic cloning techniques [3,4]. Conversely, proponents of the zoonotic origin would point out that recombination events naturally and regularly unfold with CoVs [5]. Indeed, simple evolutionary mechanisms could explain the evolution of even the out-of-frame insertion of an FCS in SARS-CoV-2. Accordingly, it has been argued that the S protein used by the Wuhan Institute of Virology, led by Dr. Shi, did not contain this site [6]. Thus, besides the suggested laboratory origin [1], the FCSs may have emerged naturally in some CoV and been maintained for their evolutionary benefit. In any case, the focus here is not on past events but on how these and related CoV features could become a future problem.

As in the biblical tale, arguing over the FCS’s origin could fuel a never-ending debate. Ironically, King Solomon did not resolve the dilemma at the same level at which it was presented. Analogously, reframing the study of the FCS insertion to a different level could reveal insights and information that may help inform future biorisk policy. King Solomon’s starting point was the *pretense* that either scenario could be true, so that both women would be equally likely to get their “share” of the baby. The analogous approach would assume, for the sake of argument, that both a natural and lab-based genesis of the sequence in question could be true and investigate the insights that can be derived, which may prove critical at a different level—emerging and future biorisk gaps. Thus, disengaging from the debate over viral origin allows analyses to reveal novel vulnerabilities that may be exploited if such a forward-looking assessment were not performed.

The subsequent analysis aims to reveal exactly these vulnerabilities. It will argue that the type of recombination events as postulated by Ambati et al. [1] fosters unrecognized biorisk potentials. Here, biorisk refers to any adverse event that research may cause or facilitate, ranging from unintended laboratory mishaps (biosafety) to the purposeful development of bioweapons (biosecurity) [7]. Accordingly, the article explores hypothetical scenarios in which the FCS could become incorporated into a CoV. While this does not constitute proof of a laboratory origin for SARS-CoV-2, the analysis provides strong evidence that synthetic mRNAs can recombine with susceptible viruses under specified lab conditions, producing harmful escape mutants with characteristics not expected for RNA viruses, such as CoVs. Because such traits are unexpected or an analysis mistakenly perceived as insinuating a specific origin for SARS-CoV-2, relevant information may be disregarded, allowing hostile parties a notable advantage in exploiting the core vulnerabilities. The article argues that, since Ambati et al.-type recombination events are almost always discussed in the context of the viral origin, their potential for future malicious exploitation may be largely overlooked.

The key hypothesis of this article is that the sequence overlap between the SARS-CoV-2 insert surrounding the FCS and the *MSH3* gene portion creates unrecognized biorisk concerns. These will be explored at several levels: (a) envisioning laboratory experiments that could, at least theoretically, engender such inserts in various CoVs; (b) describing the feasibility of such scenarios by drawing parallels from known characteristics of CoVs; and (c) depicting the novelty of these aspects and why they currently evade biorisk scrutiny. This analysis will not only reveal unrecognized biorisk gaps that could endanger future research, but will also propose how many of these concerns could be remediated.

Although Ref. [8] analyzed the feasibility of the Ambati et al. postulate and indicated foundational biorisk concerns that may ensue in related circumstances, it could not ascribe a clear function to MSH3, despite its seemingly central role in this regard. It also did not include recent discoveries about key CoV traits, including their nuclear role and capacities to subvert human DNA repair mechanisms. Moreover, relevant insights about pertinent (but still insufficient) biorisk considerations and new policies [9] had not been published.

Below, an updated evaluation of the Ambati et al. hypothesis incorporates DNA repair mechanisms, CoVs in the nucleus, oncogenic viruses, specifics of viruses in cancer research, anticancer drugs, and the complex interactions among host defenses, DDR inhibition, DNA repair, and viral escape mutants. Deliberately separating this from the viral origin question exposes the plausibility of unaccounted biorisks.

Outline: This article substantially extends the hypothesis by Ambati et al., unrelated to the context of viral origin. Instead, it discusses unappreciated biorisk vulnerabilities that evade biorisk policy and oversight.

- A vast spectrum of laboratory experiments that could result in the postulated FCS recombination event in various CoVs is described. These considerations provide the mechanistic underpinning of processes that could converge in the type of situation envisioned by Ambati and colleagues.
- Much focus is placed on RNA viruses and their nuclear role, including their hijacking of DDR processes and DNA repair. It describes how some of these attributes overlap with viruses utilized in cancer research and foster recombinant escape mutants.
- The article also considers the possibility that acquired sequences could be expressed as siRNAs. Very similar scenarios have been described during influenza virus research. An extensive host–gene knockout screen involving siRNAs transfected into cells exposed to the virus identified a related MSH gene as the most critical component for viral clearance and cell survival.
- A central concern emerges, therefore, via experiments that employ large libraries of RNAs with regulatory capacities, e.g., for the deliberate silencing of host genes during infection with (oncogenic) viruses or during chemotherapy, where the suppression of *MSH3* is a pivotal aspect.
- Informed by the complex interplay that could foster an Ambati et al.-like recombination event, specific gaps in biorisk policies are identified. Although some of these are addressed by the recently developed “Sequences of Concern” paradigm, this potent framework also does not cover several of the new vulnerabilities. Specific biorisk attributes that could enable an Ambati et al.-type event, supported by genome functionality and their combination, such as the NLS/FCS overlap in SARS-CoV-2, antisense sequences, and specific evolutionary pressure, are highlighted and extended to more general terms.
- The various indistinguishability scenarios create a theoretical bottleneck that calls for a refinement of biosafety and biosecurity principles. The article concludes with recommendations gleaned from the mechanistic underpinnings of the Ambati et al.-type scenario and those developed in related fields facing analogous challenges.

## 2. Background on CoV Recombination, the Gene Sequence Overlap Identified by Ambati et al. [1], Overlapping Functional Elements, and Key Questions

**Figure 1:**
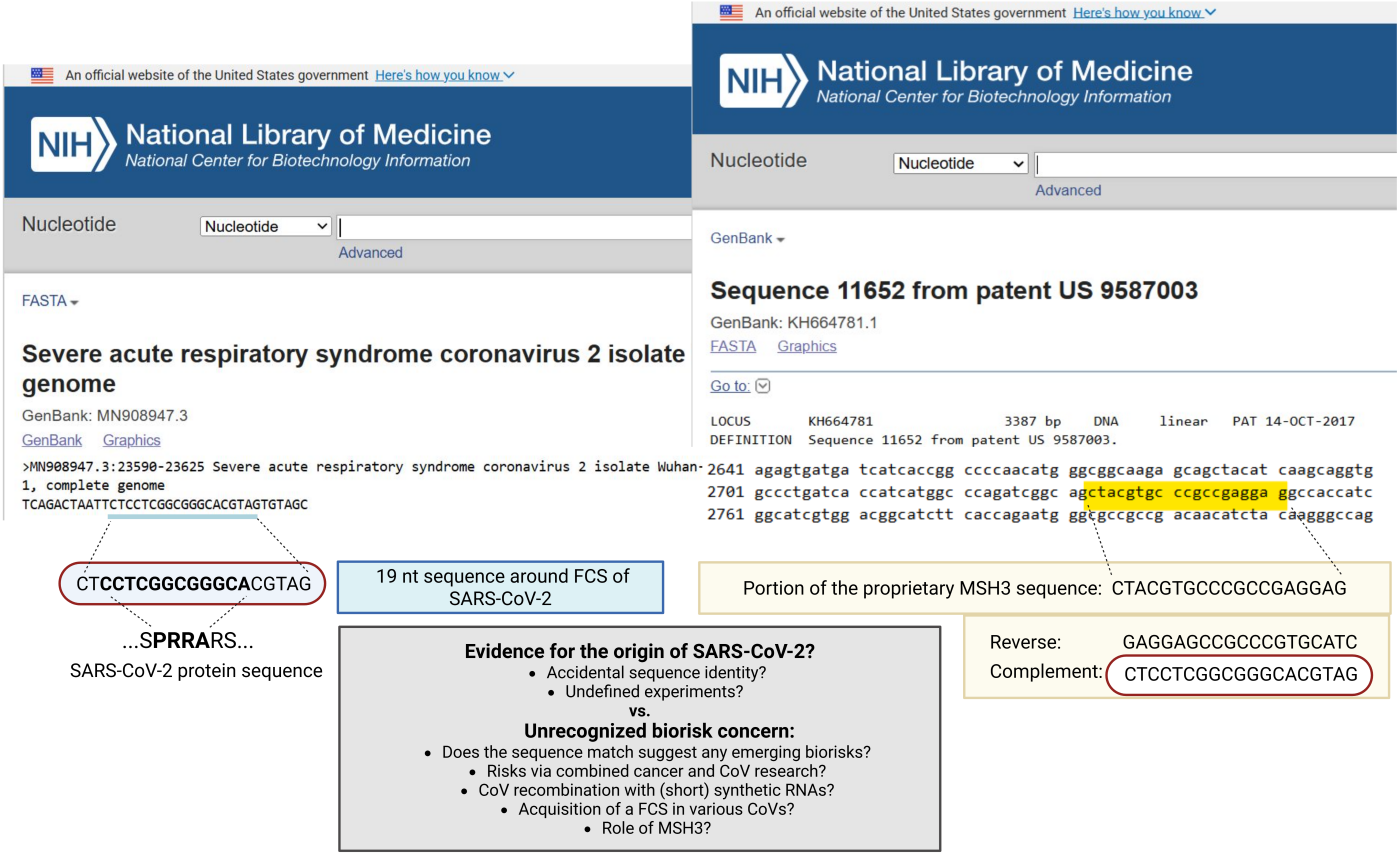
Synopsis of the sequence overlap identified by Ambati et al., which they had found via a BLAST [1] search. The left-hand side of the figure illustrates the GenBank entry of the relevant portion in the SARS-CoV-2 genome as detailed. The right-hand side depicts the corresponding portion of the sequence listing of patent US 9587003. Although this sequence identity (red ovals) is often analyzed in the context of the SARS-CoV-2 origin, this study focuses instead on previously unrecognized biorisk implications. The screenshots/excerpts from NCBI GenBank records MN908947.3 (**left**) and KH664781.1 (sequence 11652 from US Patent 9,587,003; (**right**)) is available in the public domain and reproduced here without alteration, apart from the blue underline and yellow highlight, which are added for enhanced clarity. FCS—furin cleavage site; nt—nucleotide. Created in BioRender. Mueller, S. (2026) https://BioRender.com/yc3pyzb.

In Ref. [8], a theoretical analysis was conducted to extend the observation made by Ambati and colleagues, aiming to describe the type of experiments that could result in the integration event of the FCS. In brief, this postulate is based on the observation [1] of a 19-nucleotide-long RNA sequence including the FCS which is 100% identical to the reverse complement of a proprietary sequence involving the human mutS homolog (*MSH3*). The actual sequences in question, based on their respective GenBank records, are depicted in Figure 1. It is essential to reiterate that this is not about the evolution of SARS-CoV-2 but the potential of its integration in a broad context currently not covered by DURC policies and oversight (recapitulated in Table 1). This section summarizes and updates the underpinnings of how such sequence inserts could emerge in CoVs. The following sections extend biorisk implications to cancer research more generally, and also explain the critical role of MSH3, which had previously remained unresolved. In turn, the insights suggest demanding biorisk gaps that will be further discussed in the final sections.

### 2.1. Coronavirus Recombination

In general, genetic recombination is the exchange of genetic material between different organisms. In viruses, recombination likely contributes to the emergence of new viral lineages, expansion in host tropism, adaptations to new environments, and virulence and pathogenesis. With CoVs, frequent recombination events can facilitate cross-species transmission, particularly when it occurs in the spike gene [10,11]. It is one of the primary procedures for viral rapid adaptation and evolution and the core process underlying the postulate of synthetic RNA integration in a CoV described by Ambati and colleagues.

Despite its known significance during natural viral evolution, the mechanism of recombination remains poorly understood [11]. Recombination in RNA viruses can be realized in several ways. This event is typically assumed to take place when multiple viruses simultaneously infect the same host cell and undergo genetic segment exchange [10]. This necessitates that two viruses are in the same host, in the same cell, and at the same point of replication within the cell [11]. Surprisingly, apart from the work by Ambati et al., and further analyzed in [8], there has been little attention paid to recombination between viruses and synthetic gene sequences. Since recombination resembles an evolutionary “fast-forward” by quickly shuffling genetic material between vastly different viruses (or RNA molecules), both natural recombination and that involving synthetic RNAs can produce new lineages in much shorter time than by mutation alone.

#### 2.1.1. Homologous vs. Non-Homologous Recombination

The central step of recombination in RNA viruses happens when, during RNA synthesis, the RNA-dependent RNA polymerase (RdRp) switches templates between the two parental RNAs. This mechanism is often also termed “copy-choice” because the polymerase literally “chooses” a new RNA template to copy mid-synthesis. When the template switching occurs between parental RNA molecules that are similar in sequence, depending on whether this takes place on the matching or unrelated region, it is called homologous or aberrant homologous recombination, respectively. In contrast, non-homologous recombination does not depend on sequence homology. This is possible as sequence complementarity may not be the only factor that brings the parental RNAs into proximity; other features of the RNAs, such as RdRp binding sequences, secondary structure, and heteroduplex formation between parental RNAs, can initiate and terminate the reactions and their outcome [12].

**Figure 2:**
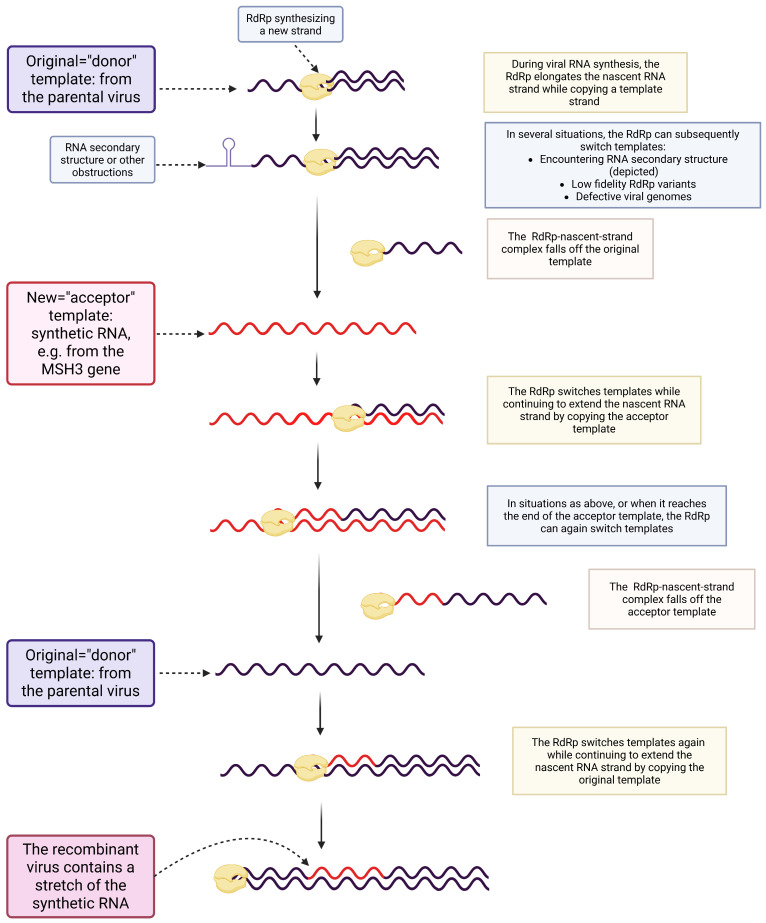
Mechanistic underpinnings of copy-choice non-homologous recombination [1,10,21]: After the RdRp begins elongating the nascent RNA strand while copying the donor template, the polymerase may slow or stall due to complex secondary structure, a mismatch, or damage in the RNA. This stalling promotes slippage or dissociation of the nascent RNA-RdRp complex from the donor RNA. Template switching (copy-choice) occurs when the RdRp–nascent strand complex, once dissociated, anneals transiently to another RNA molecule (the acceptor template). Stabilized by transient pairing (minimal complementarity), the polymerase then resumes RNA synthesis on the new template. Essentially, Ambati et al. [1] envision a template switch, whereby RdRp, while copying the viral genome, momentarily jumps onto some *MSH3*-derived gene fragment as the second template, incorporates the 19 nt stretch that encompasses the FCS, and then returns to the viral (original) template. Created in BioRender. Mueller, S. (2026) https://BioRender.com/knm17q7.

As non-homologous recombination is the process envisioned by Ambati and colleagues, the key steps, refining those provided in [1], are highlighted in Figure 2.

**Table 1:** Summary of the framework developed in [1,8].

Key Consideration	Rationale
The sequence surrounding the SARS-CoV-2 FCS, as postulated by Ambati and colleagues [1].	A 19-nucleotide sequence within the SARS-CoV-2 genome, encompassing the FCS, exhibits a 100% complementary match to the reverse complement of a codon-optimized proprietary sequence (nt 2733-2751 of SEQ ID11652, GenBank: KH664781.1, from patent US 9587003) derived from the human mutS homolog (*MSH3*) gene. This sequence CTCCTCGGCGGGCACGTAG encompasses the PRRA motif in the FCS. Ambati et al. wonder if the insert could have happened during laboratory research. Paradoxically, the match involves the reverse complement of the *MSH3* gene. Ambati et al. point out that single-stranded RNA viruses utilize negative-strand RNA templates in infected cells. They suggested that in some CoV-infected human cells overexpressing the *MSH3* gene, copy choice recombination with a negative-sense viral RNA template led to the integration of the *MSH3* negative strand into the viral genome during viral replication.
The involvement of MSH3, as first suggested by Ambati et al. [1].	SEQ ID11652 from the Moderna patent [2] is transcribed to an *MSH3* mRNA that appears to be codon optimized for humans. MSH3 is a critical component of the DNA mismatch repair (MMR) pathway. The MMR pathway is one of the main pathways of the DNA damage response (DDR), a network of complex mechanisms for DNA damage detection and repair to combat DNA-damaging agents. Importantly, as pointed out by Ambati et al. [1], overexpression of *MSH3* is known to interfere with mismatch repair. As such, they suspect that *MSH3* replacement with a codon-optimized mRNA sequence for human expression likely has applications in cancers with mismatch repair deficiencies.
The potential role of DDR agonists [8].	The main traditional pillars of cancer treatment, chemotherapy and radiotherapy, exert their anticancer activity by directly or indirectly inducing DNA damage [13]. Underlying this is the rationale that DNA damage-inducing therapies (a) prevent cancer cells from replicating, (b) cause cancer cells to undergo programmed cell death, and (c) exploit defects in cancer cell DNA repair mechanisms, which can make them more sensitive to DNA damage than normal cells. However, both healthy and cancer cells employ the DDR as a protection mechanism, as the integrity of their DNA is constantly challenged. Thereby, the deliberately induced DNA damage can be repaired in a tightly controlled fashion, counteracting the effects of DNA damage-inducing therapies and possibly even leading to therapy resistance. Understanding the processes and pathways of DNA repair in a tumor environment and therapeutic interventions is, therefore, a central question [8].
The potential role of MSH3 in DNA damage repair and cancer [8].	MSH3 and MSH2 form the MutSβ complex that recognizes double-strand DNA breaks, incl. those induced by chemotherapy drugs. MSH3 participates in DNA damage repair, specifically homologous recombination (HR) repair induced by such treatments. In turn, as MSH3 can regulate the DNA damage response and extent of apoptosis induced by chemotherapy, the status of MSH3 in tumors can influence their response to specific chemotherapy agents. For example, *MSH3*-deficient cancer cells are more susceptible to DNA-damaging drugs such as oxaliplatin and SN-38, likely due to their impaired ability to repair DNA double-strand breaks [14]. Nonetheless, the involvement of MSH3 in cancer is not straightforward, for overexpression of *MSH3* is known to interfere with mismatch repair [1]. Additionally, MSH3 is interesting for its inherent shuttling features that are regarded as “controllable” [15]. Naturally, the protein shuttles between the nucleus and cytoplasm in response to inflammatory stimuli. *MSH3* harbors two nuclear export signals (NES1 and NES2), which control its nuclear import/export. This can be modulated, as, e.g., the acetylation status of lysine residues in the *MSH3* NLS effectively controls the subcellular localization of MutSβ.
Switch the focus/language: an NLS besides an FCS [8].	An unexpected mystery surrounding SARS-CoV-2 was identified by Sattar et al. [16] in some elegant experiments, which showed the following: Both the spike (S) protein and mRNA translocate into the nucleus in SARS-CoV-2-infected cells. The nuclear translocation is mediated by a novel NLS in the S protein. This new NLS motif is present at the polybasic FCS. The novel “PRRA” FCS is subsumed within the longer sequence “PRRARSV.” Importantly, “PRRARSV” is a novel NLS. The double NLS/FCS functionality was unexpected and seems astonishing. Both sequences preserve their functionality.
Recombination involving CoVs [8].	The feasibility of such an event is underscored by fundamental features of CoV recombination. Recombination is a natural process frequently employed by CoVs. Recombination has long been thought to be dictated by the RNA donor or acceptor sequence and RNA structure. However, it has only been in recent years that determinants besides the CoV RNA sequence and structural factors have emerged. Now, it is understood that genome function, fitness, and selective pressure are the most critical in this process [17,18]. Likewise, a Bayesian analysis of CoV recombination reveals that the process is highly frequent and can boost viral fitness [19]. In this light, any evolutionary pressure imposed on a CoV as discussed above would especially favor recombinants that align with the Ambati et al. postulate. Such escape mutants would further enhance the natural capacity of CoVs to utilize a nuclear phase for their advantage. Specifically, from the viral “perspective,” escape mutants that have picked up the *MSH3* sequence would have a novel NLS, which would support their nuclear translocation. Via the double NLS/FCS feature, they would simultaneously have a novel FCS that further enhances the infectivity of the virus.

#### 2.1.2. Recombination as a Part of Replication and the Generation of Complementary Strands

Technically speaking, during recombination in RNA viruses, the polymerase moves along a donor RNA while elongating a nascent complementary strand and then jumps to an acceptor RNA and continues elongation [20]. To make the conceptual “strand switch” more obvious, replication polarity details are omitted in Figure 2, in line with other publications [1,10,21]. Doing so is also justified for the following reason: Replication of SARS-CoV-2 and other single-stranded RNA viruses with an RNA genome of positive polarity produces negative-strand RNA intermediates that are then transcribed into positive-strand RNA genomes packaged in progeny virions. Thus, as template switching first generates recombinant minus strands, and the recombinant progeny RNA genomes are produced by using the recombinant minus strands as templates, this matches the final chimeric genome depicted.

#### 2.1.3. Non-Replicative Recombination

Other forms of recombination in RNA viruses may take place as well. Co-infection of a cell by multiple viruses can cause RNA fragmentation. Subsequent cleavage and rejoining of these fragments can generate recombinant molecules in a replication-independent manner [10]. Both the replicative copy-choice model and the non-replicative breakage-rejoining model are discussed in the literature. However, there is still a lack of understanding of the exact recombination triggers and the mechanisms behind them.

### 2.2. Likelihood Estimates Versus Maintenance of Recombinants with Evolutionary Advantage

The reverse complement of the proprietary sequence in SARS-CoV-2 could be a random coincidence. Even though Ambati and collaborators calculated a low coincidence likelihood for this insert, the actual odds of such events are substantially shaped by the environment and the extent of viral adaptation to selective pressure. A commentary to their article contests their estimates, arguing that the sequence match could have been a chance occurrence alone [22].

As discussed in [8], likelihood estimates may be inappropriate, particularly in laboratory experimental settings. Some of the main points include the following:

- It has been known for thirty years that various experimental conditions can effectively trigger rapid RNA virus evolution, endowing them with potent evolutionary advantages.
- Various experimental conditions are well established to advance the natural propensity of RNA viruses for recombination.
- Recombination plays important roles in the spread, virulence, pathogenesis, and vaccine escape of viruses; for example, the emergence of novel CoVs with enhanced virulence can be explained by recombination events.
- For the same results to be produced by mutation alone, this would require long spans of time. Via recombination, CoV evolution does not unfold via a slow accumulation of adaptive mutations in a piecemeal fashion. The non-continuous process substantially complicates likelihood estimates in addition to the known defects of sequence-based measures and determinants.
- CoV recombination is a promiscuous event that is significantly influenced by evolutionary mechanisms and selection processes. The selection and propagation of recombinant mutants are driven by their replication fitness and the prevailing selection pressures.

The concept that recombination rapidly reshuffles genetic material, enabling the persistence of progeny with advantageous traits under selective conditions, is well established in studies of natural RNA virus evolution but has received limited attention in laboratory settings, where it may influence the emergence of mutants that escape experimental constraints.

### 2.3. The DNA Damage Response, DNA Repair, and Host Homologous Recombination

Essentially, a virus is a segment of foreign nucleic acid that engages host cell machinery to generate progeny viruses. Host organisms have developed sophisticated detection systems to activate antiviral responses in their defense: these are intimately linked to the DNA damage response (DDR), a complex network of signaling pathways that safeguards cellular DNA to maintain genomic integrity both during replication and when the cell is under threat from endogenous damage and exogenous agents [23]. Activation of the DDR can occur in response to the presence of viral DNA, virus-induced DNA damage, or cellular or aberrant DNA structures during viral replication. If left unresolved, DNA lesions can result in DNA strand breaks, chromosomal aberrations, genome instability, cell death, and carcinogenesis [24]. Given the high cellular cost of DNA damage, organisms have evolved multiple repair pathways, including nucleotide excision repair (NER), base excision repair (BER), and mismatch repair (MMR). *MSH3*, one of the main components of the sequence identity observed by Ambati et al., is a key MMR gene; it encodes a protein that dimerizes with MSH2, another MMR protein, and forms the MutSβ heteroduplex that is responsible for recognizing and initiating the repair of slippage mistakes at dinucleotide or longer repeats insertion–deletion loops [25].

DDR also controls the activation of cell cycle checkpoints, which prevent cells with DNA damage from progressing into mitosis. DNA damage that cannot be repaired can lead to activation of apoptotic pathways, which, when occurring before the production of infectious viral progeny, will have a detrimental effect on viral propagation [26]. Viruses have developed numerous strategies to antagonize DDR responses to avoid immune detection and ensure survival of the infected cell [24]. For example, when exposed to a substance or condition that activates the DDR (DDR agonists), viruses often respond by actively antagonizing the pathway. Remarkably, viruses are also capable of activating and engaging DDR processes. In light of the above, this is counter-intuitive. However, some viruses directly damage host DNA to hijack the activity of the repair proteins and harness them to their advantage [24].

DNA double-strand breaks (DSBs) are considered one of the most threatening types of DNA damage, and their repair is essential for preventing genomic instability and, ultimately, tumorigenesis [27]. They are repaired by three major pathways: homologous recombination (HR), nonhomologous end-joining (NHEJ), and microhomology-mediated end-joining (MMEJ).

Intriguingly, the MSH3 protein, one of the presumed key players in the Ambati et al. scenario, is, in addition to its canonical role as a mismatch repair protein, also implicated in DSB repair via HR [15]. From this perspective, this seems to make an Ambati et al.-type event even more mysterious. How could such an insert possibly be linked to a crucial host mechanism for repairing DNA double-strand breaks and maintaining genomic stability?

Thus, it is important to distinguish between CoVs that perform their own RNA-based recombination and how this affects the host’s DNA-based homologous recombination machinery. To complicate matters even further, it turns out that the sequence in question may have several different roles besides DNA repair, which functions in the nucleus. Since CoVs are RNA viruses without a compulsory nuclear phase, these observations are difficult to reconcile without assuming that their components perform multiple roles. Below, arguments will be provided that CoVs nonetheless often benefit from hijacking a variety of nuclear cell processes, despite being RNA viruses.

### 2.4. Gene Overlaps and Overlapping Functional Elements

Overlapping genes, where two or more proteins are coded for by the same nucleotide sequence, are a common feature of viruses [28]. Gene overlaps are widely recognized as a form of genome compression, enabling viruses to expand their protein repertoire without increasing genome length.

In some cases, a single nucleotide within a viral genome can contribute to the coding sequences of two or even three distinct proteins. Overlapping open-reading frames (ORFs) are also a common strategy among RNA viruses, allowing a single nucleotide stretch to encode multiple proteins with different functions. For example, in SARS-CoV, the accessory protein ORF9b is encoded in an alternative reading frame within the nucleocapsid (N) gene [29]. Several alternative-frame ORFs overlapping well characterized SARS-CoV-2 genes have been described [30], although there is some confusion about the definition of ORFs. Besides nucleotides being read in different frames or opposite strands, mutations may engender novel or extended ORFs and, again, new functions.

Overlapping genetic sequences in RNA viruses may not merely serve as a template for translation to produce different viral proteins. In multifunctional sequence overlaps, the same peptide (or nucleotide) stretch simultaneously fulfills two or more biological roles.

### 2.5. The FCS Vs. an NLS

The furin cleavage site (FCS) at the S1/S2 domain junction of the SARS-CoV-2 spike (S) glycoprotein has been extensively discussed in the context of SARS-CoV-2 origins, SARS-CoV-2 virulence, and COVID-19 pathogenicity [6,31]. The involved PRRA motif is unique among this group of CoVs [32,33]. It enhances spike cleavage by furin-like proteases, alters cell and tissue tropism, and contributes to efficient human infection and expanded host range in vitro.

Curiously, this FCS of the SARS-CoV-2 spike protein is part of a novel pat7 nuclear localization signal (NLS), 681PRRARSV687 [34]. That is, this seven-amino-acid stretch (PRRARSV) simultaneously comprises an FCS and an NLS. This example of a dual-purpose RNA/peptide element, in which a single sequence fulfills two biologically unrelated functions, will be central to the analysis below.

While the FCS is important, greater attention will be given to the NLS due to its independent functional activity. Thereby, both the spike protein and mRNA translocate into the nucleus in SARS-CoV-2-infected cells [16]. Since it is an RNA virus, this is even more surprising but is analogous to other RNA viruses that utilize the nuclear phase to their advantage. Moreover, these observations suggest a broader role for the nuclear viral phase within the Ambati et al. framework, involving cancer research and host DNA repair mechanisms, which will be explored below.

### 2.6. Guiding Questions

As emphasized in [8], from the outset, it is unclear in which research context the type of recombination event envisioned by Ambati et al. [1] could emerge. In this context, the virus would have to integrate three apparently unrelated elements: cancer research; the DDR protein MSH3 (or, more precisely, a segment that is the reverse complement of *MSH3*); and a coronavirus.

The main consideration in [8] was that a common denominator could be RNA viruses in cancer research. Oncogenic viruses often cause genomic instability, evade immune surveillance, and disrupt cell cycle control [35]. Another key component is the hijacking of DDR by RNA viruses. Based on the rationale that DNA lesions persist via virus-compromised DDR, fostering mutagenesis, several laboratory scenarios could aim to research the virus–host interplay in this particular context:

- The mapping of nuclear import/export of viral proteins.
- The development of agents that block viral hijacking of host DNA damage and repair systems.
- The utilization of a CoV as a vector to deliver novel drugs or agents, genetic material, or other aspects to examine or influence cancer development and the effectiveness of novel therapeutics and interventions.

Agents and processes that modulate nuclear localization are actively explored and used in cancer research and therapy [36,37,38]. The involvement of MSH3 is less clear. Canonically, since it is associated with DNA repair pathways and linked to MMR deficiency (dMMR) when over-expressed [1], several examples of experiments that combine the complete *MSH3* gene and viruses in cancer research, involving co-transfection of *MSH3* and a CoV into human cell lines, can be envisioned (Table 2).

However, recombination with a synthetic sequence, as postulated by Ambati et al. [1], immediately raises the question about the short length of the integrated sequence. Why would recombination only involve a 19 nt stretch of the entire *MSH3* sequence, and why the reverse complement? Specifically, if such an event involved the copy-choice mechanism, this would require two crossover events that are very close together, which may be regarded as practically unlikely [8]. Below, some potential mechanisms, while entirely speculative, that can resolve these paradoxes will be developed.

A central question addressed in the following analysis is that of how integrated sequences may confer advantages to viruses and promote their survival, particularly in contexts that fall outside current biorisk regulation and oversight. As such, it is not an expose of viral–host interactions in general but specifically of unappreciated aspects fostered by Ambati et al.-type gene integrations. Although host immunity and viral adaptation are important contributors, a comprehensive review of CoV evolution or SARS-CoV-2 pathogenicity lies beyond the scope of this article. Rather, it aims to identify unappreciated processes and potentials that could jeopardize the safety of laboratory experiments or enable their malicious subversion. The hypothesized biological mechanisms are extrapolated from known published viral traits.

In some of the later sections, the approach is reversed, whereby the identification of possible biosecurity gaps prompts the question of relevant biological features that could support unrecognized or unwanted viral evolution. Therefore, throughout the article, the host–pathogen interplay linked to biorisk considerations helps identify unappreciated aspects related to the type of recombinations envisioned by Ambati et al. [1].

## 3. Coronaviruses and the Nucleus

### 3.1. DDR Antagonism, a Double-Edged Sword: The Potential for Viral Evolution

A specific dilemma that arises in the development of cancer therapeutics that consist of DNA repair inhibitors is that they may also impact the survival and fitness of any viruses involved. If DDR is compromised (e.g., by *MSH3* overexpression or pharmacologic inhibitors), DNA lesions persist; this directly fosters mutagenesis, as well as viral adaptation. At the same time, if DNA lesions accumulate, this may trigger apoptotic programs. While these are desired in the context of cancer cells, they could also impair viral replication, triggering a complex pathogen–host interplay.

Antagonizing DNA repair may benefit RNA viruses in ways that are only beginning to be revealed [26,42]. Moreover, as the DDR network is also intimately tied to innate cellular antiviral response [44,45,46], agents that impair DNA repair may inadvertently support viral evolution and adaptation. In all, viral mutants that emerge under conditions of DDR antagonism and antiviral pressure may possess enhanced capacity to disrupt or evade host defense and subvert DNA repair pathways to their advantage.

### 3.2. Nuclear Import and Export Signals in CoVs

CoV genome replication and transcription occur in the cytoplasm, and the life cycle of CoVs does not indicate any dependency on the nucleus. Nonetheless, it is known that RNA viruses antagonize DNA repair proteins. Indeed, it has been suggested in [26] that their manipulation of components of the DDR pathway may, in analogy to DNA viruses, allow their pathogenesis and propagation. However, even in 2024, the necessary details of these processes were insufficiently understood [24].

In general, the translocation of macromolecules greater than 45–50 kDa across the nuclear pore complex is contingent upon the presence of specific peptide motifs: nuclear localization signals (NLSs) for import and nuclear export signals (NESs) for export. CoVs have several of these sequences, and in some cases, some of their proteins have been confirmed in the nucleus.

- Several CoV proteins contain NLS and/or NES and localize to the nucleus [42].
- The SARS-CoV N protein contains multiple NLSs, and its nucleolar localization was indeed observed [42].
- In addition to the S protein of SARS-CoV-2, shown to be present in the nucleus [16], many other viral proteins, when overexpressed, are nuclear. Whereas many of these proteins are small and may enter the nucleus by passive diffusion, this indicates a critical feature of these viruses [42].
- In 2020, using a bioinformatic analysis, Singh and Singh [47] reported that the S2 subunit of SARS-CoV-2 strongly interacts with the key human tumor suppressor proteins p53 and BRCA-1/2. These proteins are critical for maintaining genome integrity, regulating the cell cycle, DNA repair, and apoptosis. This computational study laid the basis for the idea that SARS-CoV-2 infection or spike protein expression might play a role in cancer-related pathways and DNA damage responses, which was validated in 2024 [43].

### 3.3. Expected Characteristics of Escape Mutants with an Improved Nuclear Presence

**Figure 3:**
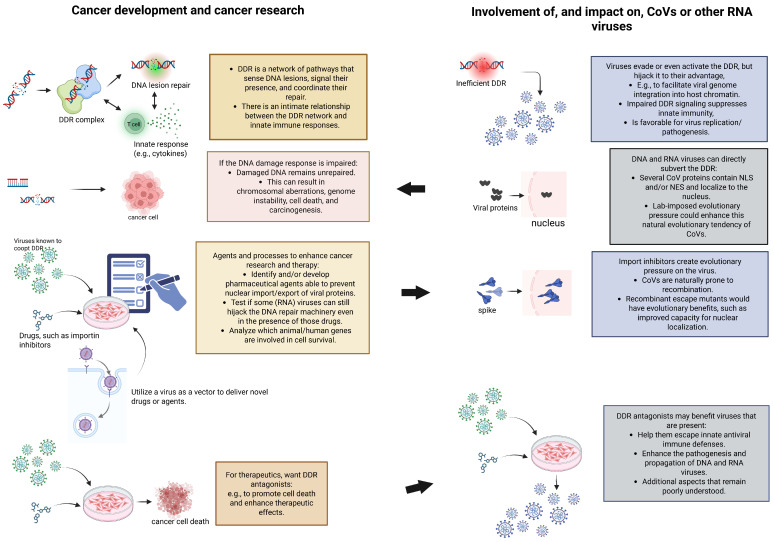
Various unrecognized lab experiments that combine cancer research with coronaviruses (CoV) or other RNA viruses may foster covert viral evolution and escape. The figure summarizes some of the feasible scenarios that could engender an FCS/NLS recombination event in various CoVs and exposes novel biorisk vulnerabilities. Additional related mechanisms and relationships that have recently emerged during experiments involving influenza, or oncogenic viruses more generally, are delineated in the sections below. Although a proportion of the underlying mechanisms are known for DNA viruses, they are mimicked by RNA viruses. As a result, susceptible CoVs could easily be endowed with related features and escape biorisk scrutiny as they are commonly not expected to share such characteristics. A deep understanding of these potentials may help deter malicious exploitation of particular laboratory experiments that, to date, fall outside of biorisk policy and oversight. DDR—DNA damage response; NLS—nuclear localization signal; NES—nuclear export signal. Created in BioRender. Mueller, S. (2026) https://BioRender.com/osrda0h.

Without going into details, several types of lab experiments related to Ambati et al.-type scenarios may involve unintentional selective pressure for viruses and could inadvertently aid viral evolution (summarized in Figure 3). If such selective pressure were present, the resulting escape mutants might display several notable characteristics:

- An NLS that improves nuclear entry of spike or other viral proteins. Indeed, from the viral “perspective,” recombinant mutants may particularly be selected and maintained for their improved capacity for nuclear translocation, which could be facilitated by a novel NLS as often acquired by CoVs.
- An enhanced FCS that boosts infectivity. Interestingly, the selective pressure that mediates the acquisition of a novel NLS may inadvertently generate an FCS. This phenomenon was demonstrated by Sattar and colleagues [16], who showed that both the spike (S) protein and mRNA translocate into the nucleus in SARS-CoV-2-infected cells. While nuclear translocation is mediated by a NLS within the S protein, this newly identified NLS motif is located at the FCS.
- Altered sensitivity to chemotherapeutics and stronger evasion of antiviral immunity.
- Ability to manipulate host DDR, cause DNA damage, or affect cell-cycle checkpoints for viral advantage.

### 3.4. Evidence of Coronaviruses Entering the Nucleus and Subverting Host Immune Processes

Most of our understanding of how viruses benefit from entering the nucleus comes from studies on DNA viruses. However, in recent years, increasing evidence has revealed similar mechanisms among RNA viruses.

#### 3.4.1. Advantages for CoVs That Enter the Nucleus

Despite primarily replicating in the cytoplasm, CoV and other RNA viruses can gain substantial advantages by entering the nucleus:

- Several human CoVs before SARS-CoV-2 (SARS-CoV-1, MERS-CoV) induce host DNA damage responses and cellular stress [42]. CoV clearance is enhanced by blocking nuclear entry, while viral infection is reduced by inhibiting nuclear export. Notably, pharmacological inhibition of nuclear export leads to nuclear accumulation of viral proteins and significantly diminishes infection [42].
- Several studies have revealed that SARS-CoV-2 can induce DNA damage, genomic instability, cell cycle deregulation, and impair DNA repair mechanisms during its replication in mammalian cells (reviewed in [24,48,49]). A separate study reported that SARS-CoV-2 infection triggers a rapid induction of the DDR, which is quickly downregulated thereafter [42]. The virus-induced DNA damage elicited an altered DNA damage response [49,50]. Curiously, the key viral proteins involved in [50] are ORF6, NSP13, and N. Although the S protein may not be directly responsible, the spike protein’s heightened nuclear translocation could indirectly support these phenomena. By hijacking the cell’s import/export machinery, it may favor the nuclear trafficking of viral proteins over host proteins.
- By entering the nucleus, viral proteins can disrupt host nuclear–cytoplasmic trafficking, leading to impaired nucleocytoplasmic transport and inhibition of innate immunity. This is well documented for various CoVs [51]. Specifically, SARS-CoV-2 Nsp1 has been reported to inhibit mRNA nuclear export, further contributing to host mRNA export inhibition and viral pathogenesis [52]. SARS-CoV-2 Orf6 positions itself within the nuclear pore complex (NPC) through interaction with the Rae1/Nup98 complex [53]. This blocks both protein import and mRNA export through the NPC, ultimately supporting viral replication within host cells.
- Nuclear localization could help the virus evade cytoplasmic innate immune sensors, shielding viral RNA and proteins from detection and degradation. The interaction of viral proteins within the nucleus (or even viral mRNA [16]) may subvert host transcriptional or critical host defense processes supporting viral persistence.
- Indirect evidence that nuclear import of viral proteins can benefit RNA viruses can also be seen in how they respond to certain drugs. Agents known to target the nuclear import pathways or that block nuclear entry of viral proteins primarily act by inhibiting host nuclear transport receptors (importins). Specifically, ivermectin is a proven inhibitor of importin-mediated nuclear transport, and several studies have demonstrated that it markedly enhances SARS-CoV-2 clearance [42].

#### 3.4.2. CoVs with Enhanced Nuclear Entry and Immune Evasion Traits Can Have a Particular Benefit in a Cancer Environment

An RNA virus with improved capacity for nuclear transport likely gains additional advantages by evading or hijacking DDR processes. Their nuclear involvement can usurp numerous processes involved in genome surveillance, repair, and antiviral defense.

Cancer cells exhibit altered nuclear transport, dysregulated signaling, and impaired antiviral immune responses. This creates a permissive environment for viruses with nuclear access to benefit infection and replication, specifically in such an environment [54]. Conceivably, RNA viruses with enhanced nuclear entry and immune evasion traits could evolve in a cancer environment, and it is expected that these viruses could be particularly harmful in cancer patients. Some of the advantages conferred by increased nuclear access could be specific to cancer cells, while others may have a broader impact on viral fitness in general.

This disparate response in cancer versus non-cancer cells has been demonstrated for SARS-CoV-2 via the interference of its spike with p53 signalling. When Zhang and El-Deiry [43] investigated the effects of transfected SARS-CoV-2 spike DNA on mammalian cell expression in cancer cells, they found that

- The SARS-CoV-2 spike protein suppressed p53 transcriptional activity in cancer cells.
- This suppression was specifically observed in the case of chemotherapy-induced activation of p53-dependent genes.
- The suppressive effect was observed even after nutlin exposure in wild-type p53-expressing cells. Nutlin compounds are useful in experimental cancer research as they specifically inhibit the interaction between the tumor suppressor protein p53 and its negative regulator, MDM2 [55]. Under normal conditions, MDM2 binds to p53 and targets it for degradation. By blocking this interaction, nutlin stabilizes and activates p53, leading to increased p53 activity in cells that have wild-type (non-mutated) p53.
- Yet, as spike interrupted the MDM2-p53 interaction, it suppressed p53’s transcription of key genes involved in cell cycle arrest or apoptosis (p21, DR5, MDM2).
- The p53 suppression resulted in increased viability and chemoresistance of spike-expressing cancer cells.

On the other hand, previous studies indicated that the spike protein can stabilize or activate p53 in some cell types and experimental contexts, apparently contradicting the above. However, prior work [56,57]

- Showed that p53 stabilization seems to be caused by cell–cell fusion or induction of reactive oxygen species (ROS), both of which are known stressors that can activate p53 pathways.
- Used normal (non-cancer) cells.
- Relied on a different experimental setup: Ref. [57] utilized SARS-CoV-2 or a pseudo-typed virus expressing spike protein rather than the transfection of a spike-expressing plasmid. Even though Ref. [56] studied fusogenicity and syncytia formation in SARS-CoV-2-infected cultures and reported increased p53 and p21 proteins, Zhang and El-Deiry [43] identified some inconsistencies, showing the disappearance of p53 and p21 in their study [56].

Therefore, the relationship between p53 and autophagy during CoV infection is complex and context-dependent [58]. Specifically, however, the spike apparently suppresses p53 transcriptional activity in cancer cells [43] but stabilizes/activates p53 in non-cancer cells [56,57].

There is no rationale to believe that SARS-CoV-2 was deliberately developed as a biological weapon to target cancer patients. Coronaviruses are generally known to manipulate the p53 pathway to inhibit autophagy and promote their own replication [58]. Nonetheless, viral proteins with nuclear access indicate novel biorisk potentials of how cancer cell pathways could be deliberately subverted. The suppression of p53 in cancer cells reflects the capacity of viruses to exploit an altered p53 regulatory environment in these cells to inhibit autophagy [59,60]. Because this is particularly the case in a cancer environment, the enhanced survival of infected cells impacts disease progression and treatment response. This effect is distinct from spike’s p53-activating effects in non-cancer cells under infection or fusion-induced stress. Zhang and El-Deiry [43] suggest that the spike, in the context of cancer cells treated with cisplatin, instills altered DNA damage sensing in the DDR pathway. The specific nature of this alteration was not described, but it will be revisited in the framework outlined below.

## 4. Viruses in Cancer Research and Gene-Silencing Experiments During RNA Virus Infection

The above does not fully explain the role of MSH3 in the Ambati et al. postulate. Whereas nuclear import can benefit RNA viruses in several ways, MSH3 plays important roles not merely in the DNA MMR pathway but also in some alternative DNA repair processes as well. To gain a better idea of whether the antisense *MSH3* sequence portion in a CoV may actually be of biological significance, this section first examines other potential biorisk gaps more generally. However, these immediately raise the question of how they could be beneficial to viruses. In turn, the scrutinized aspects not only address the virus alone, but its relationship with the host via a recently identified pivotal pathogen–host interface that, intriguingly, involves DNA MMR, seemingly in a broader function than previously appreciated. Consequently, the crosstalk via DNA MMR and DNA double-strand breaks (DSBs) adds another layer of complexity.

### 4.1. Increased Reliance on Viruses to Target Cancer

In recent years, traditional approaches in cancer genomics and the application of various therapeutic interventions have been vastly extended with the aid of viruses. For example,

- Certain viruses (oncolytic viruses—OVs) are engineered for targeted infection and intracellular proliferation within tumor cells. The aim is to provoke both innate and adaptive immune reactions in the host and to promote tumor cell death. Moreover, the ruptured tumor cells can release their progeny OVs and continue infecting the remaining tumor cells, which is thought to help continuously kill tumor cells [61,62].
- Viruses are engineered and developed as vectors for specifically delivering different genes, therapeutic agents, and immune-stimulating agents [61].
- Viruses are used to stimulate the host antitumor immune response [61].
- Viruses for cancer imaging and diagnostics: Oncolytic viruses are widely used to improve the efficacy of tumor imaging as they can be modified not only to target and replicate in tumor cells but also to carry specific reporter genes [61].
- Viruses can also be engineered to analyze how oncogenic viruses impair host processes, such as DDR. Particularly, viruses have been manufactured that artificially enhance suppression of the DNA MMR pathway [46]. Specifically, a recombinant influenza strain was created by adding microRNA sequences into an extended 3′-UTR that downregulates *MSH6* function.

### 4.2. General Biosafety and Biosecurity Concerns Involving Oncolytic Viruses

Viruses used for cancer research and drug development may be insufficiently defined as entities with their own capacities to mutate and adapt and thereby potentially become a hazard. They are often described as natural or genetically modified drugs [62] and, thereby, may overlook the potential for viral evolution and escape. Their possible biorisks [63] have only recently come under consideration (https://monitor.cntrarmscontrol.org/en/2024/dual-use-risks-of-oncolytic-virus-engineering/, last accessed on 17 January 2026). Specific adverse effects and risks in this context have been described, including uncontrolled viral replication, possible transmission to patients’ contacts, latent infection, and long-term adverse events, unintended mutations, and reversion to pathogenic forms [63,64]. Of great concern in the context of the potential FCS acquisition by a CoV during some lab work are the following:

- A well established method for designing oncolytic viruses consists of “Directed Evolution” [65]. With this approach, viral diversity can be increased by pooling an array of serotypes and then passaging the pools under specific conditions. Indeed, aiming to facilitate the utilization of these viruses, these conditions are often precisely those that invite recombination events. Via this method, a novel chimeric oncolytic virus was already created in 2008 [65]. However, as the focus is usually to increase drug potency and selectivity of specific cancer cells, this cannot exclude unanticipated recombinants with off-target or adverse effects. Li et al. [63] even fear that viral shedding could cause homologous recombination between an oncolytic virus and a residual wild-type virus. Nonetheless, the potential for recombination with synthetic genetic material, the core of the Ambati et al. postulate, does not seem to have been considered. Furthermore, as OVs are intended to spread robustly between tumor cells, recombinant mutations could be considerably harmful as they are often associated with enhanced viral fitness and pathogenicity [17,18,19]. These adaptations may also involve enhanced tissue tropism or the capacity to disseminate to close contacts.
- The application of viruses to deliver genetic or bioactive cargo is particularly concerning as it (a) could unwittingly endow viruses with unrecognized biological activities (such as with the unanticipated double FCS/NLS functionality) and (b) create an environment that places evolutionary pressure on the virus; (c) if escaped, these viruses may more effectively evade host immune defenses.
- The pursuit to stimulate the host antitumor immune response via viruses raises the question of variable, disparate immune profiles encountered in different contexts. Additionally, as viruses can exploit cancer-specific defects, increase genetic variation, and alter the tumor microenvironment and immune signaling, this creates non-intuitive consequences for viral persistence and cancer progression [66,67]. As a result, this may engender the opposite effects than intended, fostering viral evolution and escape.
- The concern with viruses for imaging is their systemic distribution, often performed on healthy subjects as diagnostics or in a specific cancer microenvironment. Again, a specialized oncogenic niche and immune impairment could foster viral persistence and drive such viruses to unintended adaptations, especially in a context that supports frequent recombination, as is the case with CoVs.

### 4.3. Viruses in the Context of siRNA Knockdown—Analogous Scenarios to the Ambati et al. [1] Postulate

Identifying genes that support viral survival, e.g., in cancers but also during infection more generally, has become increasingly dependent on experiments involving small interfering RNAs (siRNAs) or micro RNAs (miRNAs) [46]. siRNAs and miRNAs are both key molecules in the RNA interference (RNAi) pathway, a fundamental biological process that regulates gene expression by silencing specific mRNA molecules.

Essentially, siRNAs direct the cleavage of mRNA transcripts that contain full sequence complementarity, whereas miRNAs interact with transcripts possessing partial complementarity. A clear distinction between siRNAs and miRNAs is difficult, and, as their biologies overlap, these terms are often used inconsistently [68]. Both derive from dsRNA precursors. Despite their different processing, si/miRNAs function as single-stranded RNAs within the RNA-induced silencing complex (RISC), where they bind to target mRNAs via (partial) complementarity, leading to translational repression or mRNA degradation. Such regulatory RNAs will play an important role in the processes described below.

Importantly, siRNA transfection of cells of interest is often coupled with viral infection to measure RNA and protein levels of targeted genes. Informed by the Ambati et al. postulate, this reveals some unrecognized biorisk potentials in this context.

#### 4.3.1. Silencing of the DNA MMR During Influenza Infection via Chimeric Viruses

Chambers et al. [46] employed a multi-tiered experimental framework combining in vitro cell culture models, loss-of-function screening, functional validation assays, and in vivo mouse infection models to identify cell survival after influenza A virus (IAV) infection. Via silencing experiments that predominantly utilized siRNA-mediated knockdown in a club cell model, the authors identified the DNA mismatch repair (MMR) pathway as critical for enabling these cells to clear the virus without lysis, repair oxidative DNA damage induced by the infection, and mount an effective innate antiviral response. Their findings reveal unexpected relationships between IAV infection and the host DNA repair system. Additionally, such types of experiments establish a direct relationship between short synthetic RNAs and viruses, which does not seem to have received adequate biorisk scrutiny.

#### 4.3.2. Demonstration of the Importance of DNA MMR

- Chambers et al. performed a loss-of-function siRNA (small interfering RNA) screen targeting human genes involved in DNA repair, oxidative stress, and genome stability. This involved a total of 23,349 siRNAs targeting 7783 genes.
- The basic step of the experiments in Ref. [46] closely resembles the situation considered in this article. Cells containing a reporter gene were transfected with siRNAs and then infected with an RNA virus.
- In Ref. [46], the enormous siRNA library allowed systematic knockdown of thousands of genes in H441 cells. When followed by infection and automated survival readout, this enabled the recognition of host genes that are crucial for epithelial cell survival after influenza infection.
- The experimental setup, by targeting viral genes or host factors essential for viral replication, may create an environment that could drive the emergence of escape mutants. The authors accounted for this possibility, e.g., by measuring viral fitness and disease outcomes in different animal models with varying degrees of MMR suppression.

In Ref. [46], these gene knockout experiments allowed the identification of genes required by some cells to clear and survive IAV infection. Interestingly, the list of the top 15 genes included *MSH6*, the mutS homolog 6, traditionally known to be essential for DNA mismatch recognition and repair. This means that the DDR system plays an extended role and is also critical for the host’s innate response after influenza infection. Chambers et al. cannot make sense of why this gene might be involved and ask, “As an RNA virus, IAV infection is not generally thought to affect host DNA metabolic processes, and thus it was unclear why this gene would be required for cellular survival from IAV infection.” Notably, follow-up experiments confirmed that DNA MMR is essential for the survival of the targeted cells, and, significantly, for the repair of ROS-induced DNA damage during IAV infection.

#### 4.3.3. In Vivo RNAi Screening via Chimeric Viruses

A key experimental tool in Ref. [46] was the use of engineered chimeric influenza viruses to artificially suppress the DNA MMR pathway in infected cells via microRNAs (miRNAs)/small interfering RNAs (siRNAs). They did this by engineering artificial microRNAs (MSH6-amiRNA) specifically designed to knock down MMR activity into an extension of the IAV 3′ UTR.

The silencing of host processes via the engineered IAV is based on techniques known as in vivo RNAi via chimeric viruses engineered to target and suppress host mRNAs [69]. In contrast to traditional RNAi techniques, which rely on exogenous siRNA delivery, such approaches leverage replication-competent RNA viruses for the delivery of siRNAs in a physiological infection context. Specifically, the integration of these sequences into the IAV genome led to their expression as small RNA molecules, which were functionally equivalent to MSH6 siRNAs. Reduction in *MSH6* RNA levels was confirmed in mouse epithelial cells infected with the MSH6-amiRNA virus, demonstrating effective gene knockdown via this in vivo RNAi methodology.

### 4.4. Is the DNA MMR Repair System Also a Key Host–Pathogen Interface for CoV Infection?

It is becoming increasingly evident that a range of pathogens subvert the host DNA repair pathway for their advantage. Many DNA viruses and bacterial pathogens that downregulate these pathways have been described. The fact that DNA MMR is required for IAV clearance [46] is significant in that it reveals its pivotal role during RNA viral infection. Since this DNA repair pathway is responsible for excising and repairing mismatched nucleotides that arise during DNA replication or certain DNA damage, these nuclear processes targeted by RNA viruses are unexpected. This prompts the question of whether analogous processes could apply to CoVs.

#### 4.4.1. The MMR Pathway Is Required for Viral Clearance—Prolonged SARS-CoV-2 Persistence in a dMMR Context

As it turns out, MMR is essential for viral clearance of various RNA viruses, including influenza and other coronaviridae. Specifically, it seems to extend to SARS-CoV-2, as supported by the report of a cancer patient with Lynch syndrome who manifested SARS-CoV-2 PCR positivity for at least 54 days after contracting mild COVID-19 illness [45]. Patients with Lynch syndrome have deficient mismatch repair (dMMR) due to an inherited genetic mutation, and dMMR could contribute to prolonged SARS-CoV-2 survival. In the study, PCR positivity at day 54 was associated with a CT of 33.4. Even though this does not confirm survival of the virus at that point [70], it supports the notion of prolonged presence of (targeted) viral proteins.

Interestingly, Lynch syndrome tumors have highly mutated genomes and substantial immune infiltration due to somatic mutations and neoantigen loads, which probably results in stronger immunoreactions [71]. This immune-rich environment could drive antiviral responses and counteract viral infections. Persistent infection by SARS-CoV-2 in a Lynch syndrome patient with dMMR could signal that the virus has adapted a unique nuclear trafficking mechanism combined with a unique immune evasion ability.

#### 4.4.2. Deliberate Generation of a CoV to Induce Suppression of Some DNA MMR Pathways?

To unravel the influence of DNA MMR on CoVs, it seems feasible that in vivo RNAi might be engineered into CoVs just as for IAV. This section does not assert that such a process actually took place during the genesis of SARS-CoV-2. Indeed, it reveals the challenges of such an approach. The first question is which MMR genes would make sense to be targeted. Indeed, in Ref. [46], the authors had first validated the importance of the *MSH6* gene before inserting an MSH6-amiRNA into a chimeric IAV.

One of the paradoxical situations of the homology postulated by Ambati et al. concerns the reverse complement of *MSH3*. One may note that this makes this insert a potential candidate to silence *MSH3.* Nonetheless, the question is, why MSH3 rather than MSH6? As indicated below, MSH3 may not play the same important role as MSH6. Of course, this may not impede such experiments, but it also hints at the inherent problem. Countless other genes that may be central in the CoV–host interplay related to DNA repair could be targeted. Further, it is not entirely clear which short RNA could silence *MSH3.* If every possible short RNA complementary to some *MSH3* gene portion were integrated into a CoV to engineer a chimeric virus to examine their effect on the host MMR pathway, such experiments might just be much too costly.

### 4.5. In Vitro RNAi Screens and the Concern of CoV Recombination

Methods that use viral vectors to deliver siRNAs directly into animals have obvious advantages, e.g., when examining systemic physiology and inferring indirect regulators that are better captured in vivo. Nonetheless, due to their high cost, low throughput, and ethical/logistical constraints, initial discoveries of relevant MMR genes after CoV infection are not conducive to such techniques and benefit from in vitro RNAi instead.

In vitro studies also have the advantage that they can be automated, allowing for high-throughput transfection. In large siRNA screens, the most optimal siRNAs do not have to be known a priori but can contain numerous feasible candidates based on bioinformatics prediction.

Nonetheless, it does not seem that large-scale experiments targeting host–gene silencing via high-throughput siRNA screening have been scrutinized for their biorisk concerns. Specifically, to study the effect of *MSH3* suppression on CoV–host cell interaction, such experiments often involve virus-siRNA co-transfection, which facilitates their proximity and recombination. This raises the concern that some siRNAs could be permanently integrated into the viral genome, just as intentionally performed by Chambers et al. [46].

Specifically, the sequence CTCCTCGGCGGGCACGTAG (Figure 1), due to its short length (19 ntd) and seeming lack of self-complementarity, does not seem to be conducive to the hairpin formation during the formation of precursor miRNAs. However, it is conceivable that it exerts its function directly as a regulatory RNA (e.g., a mature miRNA or siRNA) through base pairing with complementary mRNA targets, such as *MSH3*’s portion GAGGAGCCGCCCGTGCATC.

The integration of some siRNAs into a CoV may or may not create a viable virus. However, as seen from the postulated Ambati et al. scenario, the sequence inserted to silence *MSH3* may have an unrecognized second function (here, as an FCS and an NLS) which could support the survival of the new virus. Replication-permissive in vitro systems could foster viral adaptation by selecting for mutants optimized for survival, especially under stress. In DNA repair-deficient cells, they could also replicate more efficiently due to relaxed cell cycle checkpoints.

**Figure 4:**
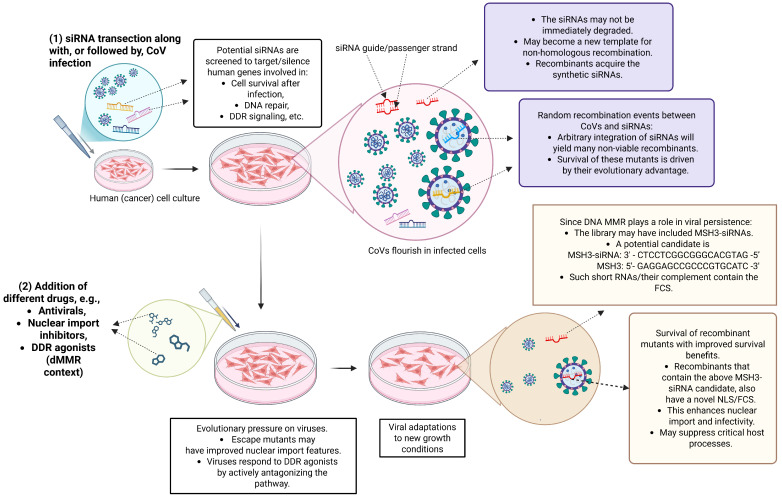
Gene-silencing experiments involving large siRNA libraries in the context of CoVs may involve unappreciated biorisks. Loss-of-function screening frequently relies on the transfection of countless synthetic short RNAs that aim to target critical host genes involved in viral infection. In this context, experiments investigating the impact of viral infection, pharmacological interventions, immunologic pathways, and immune responses inadvertently result in the co-transfection of numerous short RNAs into the same cells subsequently exposed to the virus. CoVs, in particular, are naturally prone to recombinanation. Arbitrary recombinants between CoVs and some of the candidate siRNA passenger/guide strands may not yield viable mutants (top panel). However, the selection and retention of recombination events are predominantly shaped by their evolutionary benefits (Table 1). Consequently, when these recombinant mutants are additionally exposed to lab-imposed pressure, e.g., when analyzing the effect of antivirals or viral nuclear import inhibitors, this could drive the evolution of unaccounted escape variants. Unless strictly controlled and counteracted, such unappreciated CoV features may be maliciously exploited. This situation was motivated by the FCS insert in SARS-CoV-2. Whilst not related to the origin of the virus, such types of experiments could explain why integration events, such as those envisioned by Ambati et al. could only involve a very short stretch of the *MSH3* sequence (bottom panel), something that prior work could not account for [1,8]. dMMR—mismatch repair deficiency; DDR—DNA damage response; siRNA—small interfering RNA. Created in BioRender. Mueller, S. (2026) https://BioRender.com/aap7tj9.

Combined, this suggests that co-transfection of siRNAs and CoVs into cell cultures explicitly increases the availability of short RNA fragments and drastically extends the recombination potential compared to other experiments that combine MSH3 and viruses in cancer research (Table 2). This type of setup may create the necessary environment to foster the recombination with a presumed or actual MSH3-siRNA, an antisense strand that is the perfect reverse complement of the targeted 19 ntd sequence discussed by Ambati et al. [1]. Since these factors are not widely known, such experiments could drive the evolution of clandestine recombinants (Figure 4) and lend themselves to malicious exploitation.

## 5. Potential Impact of an Ambati et al.-Type Sequence Homology on DSB DNA Repair and SARS-CoV-2 Evolution

The Ambati et al. postulate hinges on the sequence overlap between the 19 nt sequence encompassing the FCS and the reverse of an *MSH3* sequence portion. Ref. [8] warned that CoV studies targeting *MSH3* could introduce unforeseen biorisk issues, yet it failed to establish a specific function for MSH3 in the context of Ambati et al.-type experiments (Table 2). Likewise, the above also could not entirely explain the role of MSH3.

MSH3 is best known for its canonical function in MMR [25]. What is less appreciated is that MSH3 is also involved in double-strand break (DSB) repair via homologous recombination ([15] and references therein). This section envisions its essential role in an Ambati et al.-type scenario, inspired by related findings about the virus–DSB repair interplay.

### 5.1. SARS-CoV-2 and Homologous Recombination (HR)

Surprisingly, as recently demonstrated, SARS-CoV-2 seems to be able to hijack the HR machinery and subvert it for its advantage [72]. The focus of Pham et al. is RAD51, a key factor involved in HR. Whereas MSH3 and RAD51 both participate in HR-mediated repair of double-strand breaks, they act at different steps and through very different biochemical activities.

- Interestingly, despite its central role in DNA repair and anticipated nuclear localization, Pham et al. found that RAD51 accumulated in the cytoplasm of SARS-CoV-2-infected cells.
- Silencing of RAD51 impaired SARS-CoV-2 propagation. As the RAD51 protein co-localized with replicating viral RNA, these findings strongly indicate that SARS-CoV-2 exploits host cellular RAD51 to promote viral propagation.
- An immediate consequence of this proposition is that RAD51 inhibition may serve as a novel therapeutic agent for the treatment of COVID-19. The study found that multiple RAD51 inhibitors provided antiviral activities against SARS-CoV-2 both in vitro and in the Syrian hamster model.

### 5.2. Could SARS-CoV-2 Potentially Hijack HR via MSH3?

According to Pham et al. [72], SARS-CoV-2 usurps RAD51 to aid its propagation. Notably, it does so in the cytoplasm, possibly facilitated by leaky membranes in heavily infected cells or impaired nuclear import due to viral cytopathology. Conversely, via its NLS, the virus likely also hijacks host nuclear processes. Furthermore, *MSH3* contains Nuclear Localization and Export Signals and seems to be a shuttling protein that reversibly exits from the nucleus to the cytosol in response to proinflammatory signals [15]. This blurring between the nucleus and the cytoplasm raises an interesting question: does SARS-CoV-2 have the potential to use the integrated sequence reverse complement to *MSH3* to suppress or subvert DNA DBS repair and HR processes in more than one way, or could it evolve to have this capacity?

The feasibility of this concept aligns with emerging evidence indicating a functional interconnection and crosstalk between homologous recombination repair (HRR) and MMR [73]. Additionally, from an evolutionary perspective, it is feasible that key pathway proteins, such as MSH3, are utilized by more than one repair pathway [27].

#### 5.2.1. Could the Reverse Complement to *MSH3*, When Expressed, Silence Host MMR Processes?

Chambers et al. [46] demonstrated how *MSH6* can be silenced by the integrated sequence with perfect complementarity to *MSH6*. Although they established this on the influenza virus, the presence of the reverse sequence portion of *MSH3* in SARS-CoV-2 prompts the question of whether this virus might be on a similar trajectory to express functional siRNAs and silence *MSH3*. More precisely,

1. Pivotal for the experiments by Chambers et al. is the finding that viruses with essentially anti-MDA6 siRNAs in their genome prevented club cell survival and increased the severity of the disease. In other words, the integration of these sequences in the engineered viruses enabled the virus to silence the host MMR cellular survival and antiviral response.
2. Now, if the insertion of the anti-MDA6 siRNAs in IAV increased the survival and pathogenicity of this virus, it is tempting to ask if the analogous situation could apply to SARS-CoV-2 via its 19 nt sequence insert that is complementary to *MSH3*? Alternatively, one may wonder whether specific mutations in this genome portion could further enhance the capacity of future variants to more effectively target *MSH3* and induce *MSH3* silencing with notable clinical effects.

Despite this seeming analogy, there is no evidence that the sequence surrounding the FCS, if expressed as an siRNA targeting *MSH3*, has the same pivotal function as *MSH6.* Indeed, inactivation of *MSH3* does not abolish MMR activity entirely but induces microsatellite instability targeting specifically tetranucleotide repeats (EMAST) [15]. This partial impairment of MMR is also congruent with the prolonged persistence of SARS-CoV-2 in a Lynch syndrome context where dMMR is already present. However, this involves the loss of different MMR genes, such as *MSH2* or *MSH6* (https://www.ncbi.nlm.nih.gov/books/NBK1211/, accessed on 17 January 2026). Thus, reduction in *MSH3* function does not seem to impair DDR processes or benefit the virus the same way as via *MSH6*, where *MSH6* silencing alone proves sufficient to abrogate the innate immune response against IAV.

#### 5.2.2. The Non-Canonical but Critical Role of MSH3 in HR

On the other hand, beyond its necessity during classical mismatch repair, MSH3 is also implicated in HR. Interestingly, contrary to its lesser role in MMR, knockdown of *MSH3* reduces correct HR, indicating its substantial impact on the process. Specifically,

- MSH3 is involved in DSB repair through HR, unique among the MMR proteins.
- HR is mostly active during S- and G2-phases when sister chromatids are available to serve as the template during the repair to facilitate proper repair. This is unlike nonhomologous end-joining (NHEJ) repair, which is more error-prone and used when there is no sister chromatid available [25].
- MSH2-MSH3 also inhibits access of POLθ, which promotes polymerase θ-mediated end-joining (TMEJ), also known as microhomology-mediated end-joining (MMEJ), another major DSB repair pathway, which, however, is also more error-prone than HR [27].
- Based on their ability to recognize mismatched DNA sequences, MSH2-MSH3 has also been suggested to reject invading strands with imperfectly matched template DNA to prevent recombination between divergent DNA sequences. [27].
- Importantly, *MSH3* deficiency suppresses HR that repairs DSBs in an essentially error-free manner [14].
- When *MSH2* or *MSH3* is depleted, error-prone processes for DSB repair via TMEJ and NHEJ are enhanced [25].

#### 5.2.3. Error-Prone DSB Repair Processes May Be Advantageous to Viruses

Several ways in which viruses could benefit from host DNA DSB repair pathways not working perfectly—as would be the case when *MSH3* is suppressed—can be envisioned. While standard RNA virus recombination is a distinct, RdRp-driven process, utilizing the host DSB repair machinery could afford several evolutionary advantages. For example, co-opted DSB repair pathways could be engaged by the virus to make its RNA a substrate for the host’s DNA repair proteins. Since RNA is a relatively unstable molecule and susceptible to damage, this could repair viral rather than host genomes. The subversion of host HR pathways helps viruses gain control over recombination processes to support genome plasticity, adaptation, and successful propagation in diverse environments, which is a general strategy exploited by several pathogens [74].

Collectively, this suggests that the integrated sequence in SARS-CoV-2 or some of its mutants may have the potential to be expressed as a functional siRNA targeting *MSH3.* With reduced *MSH3,* error-free HR is blocked, and error-prone DSB repair pathways could be subverted for the viral benefit.

**Figure 5:**
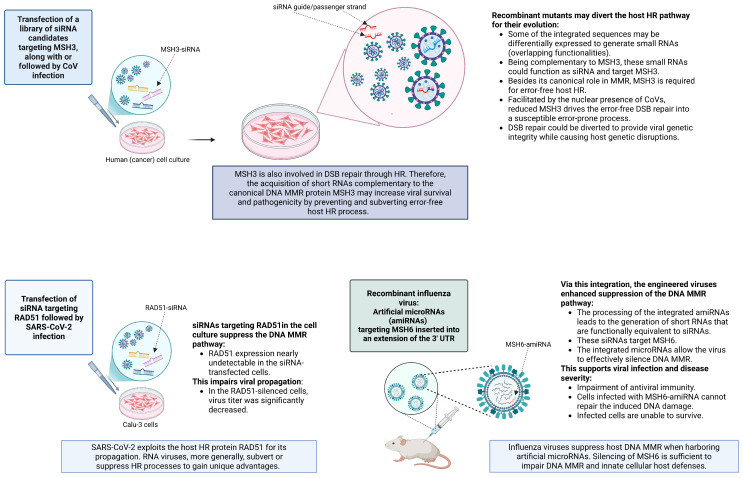
Can the co-transfection of CoVs and short RNAs complementary to host genes create recombinant mutants able to silence these genes? Top: Some of the laboratory experiments described above, via the co-transfection of CoVs and certain siRNAs, may explain why these viruses might acquire such short RNAs, as inspired by the Ambati et al. postulate. However, these events and their implications remain speculative. They are supported by the sequence overlap as described by Ambati and colleagues, which may be coincidental. Alternatively, the overlapping sequences could possess yet-unidentified functions. Specifically, research on RNA viruses has demonstrated several ways in which they suppress or hijack host DNA damage and repair processes. The observed sequence overlap in SARS-CoV-2 with the reverse of a short *MSH3* sequence raises the question of whether this homology could generate RNAs that act as siRNAs and silence this critical gene, thereby impairing its function in homologous recombination. Although there is currently no demonstration that SARS-CoV-2 exhibits this characteristic, the exact same mechanisms are (a) exploited for the engineering of in vivo RNAi processes via chimeric viruses, (b) biological plausibility. Bottom: The figure depicts two related scenarios of how RNA viruses harness host DNA repair pathways to their advantage: left: Pham et al. [72] demonstrated through in vitro transfection that silencing of RAD51 significantly decreased virus titer, in line with the proposition that this protein has been subverted by the virus for its benefit; right: Chambers et al. [46] revealed that chimeric IAV engineered to silence MDA5 enhanced its survival and pathogenicity. mi/siRNAs—micro/short interfering RNAs; amiRNAs—artificial microRNAs. Created in BioRender. Mueller, S. (2026) https://BioRender.com/cxjlhgz.

Figure 5 summarizes the three scenarios considered above, of how RNA viruses suppress or utilize host DNA repair. Whereas the notion that some SARS-CoV-2 variants may have the potential to suppress *MSH3* and its role in HR for their benefit is entirely speculative, it is based on (a) analogous processes involving in vivo RNAi, which is pursued as a versatile silencing tool, and (b) the biologic feasibility of *MSH3* silencing involving HR.

### 5.3. May *MSH3* Deficiency Drive Viral Evolution and Escape?

The above raises the following conundrum: If a CoV evokes the reduction of MSH3, thereby promoting error-prone repair processes, the accumulation of DNA damage would eventually trigger apoptotic programs, possibly impairing the virus in its replication cycle. These considerations complement those raised by Zhang and El-Deiry [43] above. Below, a theoretical explanation is provided that may help resolve both of these seeming paradoxes.

#### 5.3.1. SARS-CoV-2 and Its Paradoxical Suppression of p53 in Cancer Cells

As noted above, the conundrum raised in [43] involves the seemingly unique action of SARS-CoV-2 on cancer cells:

1. The spike protein interrupts p53-MDM2 protein interaction.
2. The suppression of p53 occurs even in the presence of chemotherapy (e.g., cisplatin), which normally induces p53 activation.
3. Cisplatin-treated tumor cells expressing spike have increased cell viability as compared to control cells.

#### 5.3.2. MSH3 and Anticancer Drugs

*MSH3* inhibitors have long been used as a tool in cancer therapy. Indeed, MSH3 is a central factor that regulates the extent of apoptosis induced by chemotherapy [14]. This is directly tied to its capacity to modulate DSB repair:

- Cytotoxic drugs, such as cisplatin, cause DNA lesions, such as interstrand cross-links (ICL), leading to the inhibition of DNA synthesis and cell growth [75].
- In tumors with existing deficiencies in DNA repair, cells are unable to adequately repair the cisplatin-induced DNA damage, exacerbating the instability of the genome. This process ultimately triggers apoptosis, causing cancer cells to die.
- The DNA repair deficiency is directly linked to *MSH3*. Whereas MSH3, in complex with MSH2, recognizes the cisplatin-generated ICLs and promotes the repair of the resulting DSBs, *MSH3* deficiency suppresses HR that repairs DSBs. Thereby, *MSH3* status can determine the extent of apoptosis and cytotoxicity of anticancer drugs.
- Notably, *MSH3* inhibition can occur via multiple pathways, such as siRNAs targeting *MSH3.*

Now, when *MSH3* deficiency increases apoptosis due to compromised DNA repair capacity in response to cisplatin, this can directly interfere with viral replication. This prompts the question of whether the disparate effect described by Zhang and El-Deiry [43] might depict a viral escape strategy in specific environments.

#### 5.3.3. Is p53 Inhibition in Cancer Cells a Viral Escape Strategy to Responses Evoked by Its HR Subversion?

Zhang and El-Deiry [43] note that in non-cancer cells, the spike does not disrupt p53. This seems to make sense as in normal cells, basal DNA damage is low, and p53 remains largely inactive. By contrast, cancer cells experience chronic replication stress and accumulate DSBs, especially after cisplatin treatment. Under these conditions, p53 is robustly activated and eventually triggers apoptosis.

It seems possible that the suppression of p53 in spike-expressing cancer cells is a viral escape to avoid p53-driven death, to allow the virus to complete its replication cycle. By contrast, in non-cancer cells, the spike protein mediates host cell infection and cell–cell fusion that causes stabilization of p53, possibly indicating the disparate responses relative to the accumulation of unrepaired DNA lesions, including those caused by the virus. While this is entirely speculative, it could also explain how the virus tackles the double-edged sword of expressing siRNAs to target *MSH3*, which could trigger antiviral responses. Its suppression of p53 can prevent the host cell from undergoing apoptosis or cell cycle arrest.

Nevertheless, this hypothesized characteristic of SARS-CoV-2 is not intended to imply a laboratory origin—for example, through chemotherapy-related experiments—but rather highlights the many unanswered questions and viral potentials that can emerge from its multiple functional sequences. Whereas the FCS has garnered substantial attention, the overlapping NLS is well confirmed but has received comparatively little attention. Beyond this, the sequence homology with *MSH3* may enable yet another function via the expression of siRNAs. These combined features alone, entirely afforded by the multifunctionalities of the overlapping sequences, could provide the basis of how such a virus may enter the nucleus and hijack host HR. It will not directly explain how the spike manages to suppress p53, feasibly for keeping the infected cell alive long enough for viral production, but only when there is an imminent threat of apoptosis. The hypotheses outlined here concerning the potential hijacking of host DNA damage response and repair by SARS-CoV-2 warrant further study to characterize these interactions overall, including their implications in cancer biology.

### 5.4. Potential Biological and Biorisk Implications of the Putative MSH3-siRNA

The above analysis examined how the sequence overlap identified by Ambati and colleagues might be relevant from a biorisk perspective about related events. Analyzing the feasibility of such scenarios has led to the investigation of how known viral traits could engender unrecognized biorisk gaps. Conversely, unappreciated technical issues not covered by biorisk policy prompted the question of how the integration of a sequence with a particular RNAi could benefit the virus. This has raised the possibility that a virus with such an insert might be able to co-opt the host HR process. Whereas this proposition does not seem to have been considered before, it may complement some bizarre SARS-CoV-2 features in cancer patients undergoing chemotherapy.

Combined, these scenarios suggest aspects of downstream effects beyond the direct impact of the insert on the DNA repair system. Although the analysis of the Ambati et al.-type scenario was largely done through the lens of cancer research, it did not consider the relationship between the virus and tumorigenesis. It appears unlikely that viral recombination patterns or their maintenance are influenced by the viruses’ potential to induce cancer. Given that cellular transformation and oncogenesis are multistep processes that develop gradually, this timescale seems too prolonged to determine which rapidly emerging recombinant variants are retained.

Nonetheless, if SARS-CoV-2 were capable of hijacking the host DNA repair machinery under certain conditions, it would be reasonable to expect an accumulation of DNA damage detrimental to the host. The above situation is a good example of the complex host–pathogen interplay. It suggests how a putative subversion of HR by the virus could evoke alternative DNA repair processes, and, only when these are lacking or overwhelmed, engage p53 to provide time for DNA repair or, if damage is irreparable, apoptosis. In such cases, p53 activation plays a central role in the antiviral response, a pathway that SARS-CoV-2 appears to hijack to facilitate its persistence and replication.

**Figure 6:**
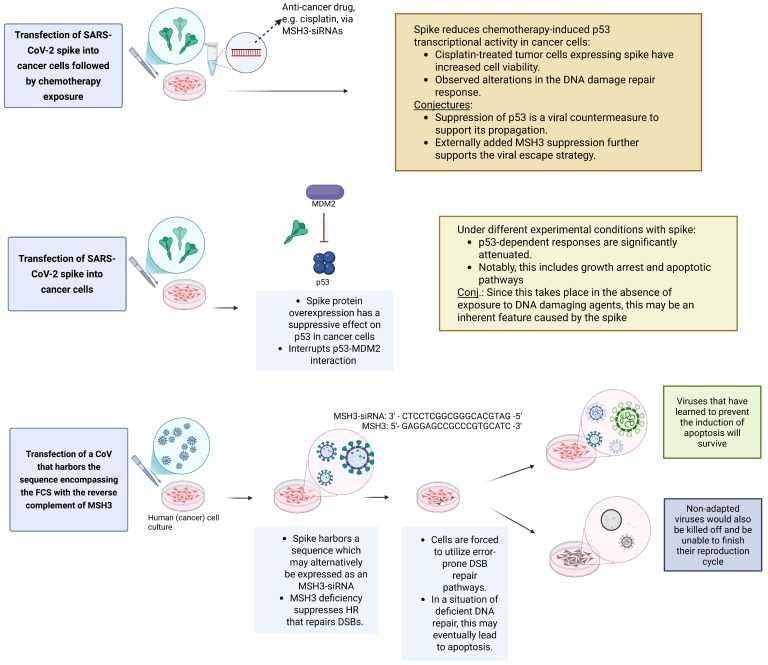
Top and Middle: A sobering observation made by Zhang and El-Deiry is that the SARS-CoV-2 spike perturbs p53 in cancer cells. This suppression was observed even after chemotherapy exposure, which would normally induce p53. The disruption of p53 without chemotherapy agents, albeit with an increase in the survival of cancer cells expressing spike, strongly suggests a spike-induced evasion strategy that may be enhanced through agents such as cisplatin. The increased combined effect raises the possibility that spike may intrinsically harbor a certain potential that analogously gets activated by cancer drugs. Although Zhang and El-Deiry could not elucidate the precise mechanisms, they noted irregularities in the host DNA damage repair response. Bottom: A virus with the sequence homology described by Ambati et al. (Figure 1) might, in theory, possess traits that fit this model. Such a virus could express siRNAs targeting *MSH3,* thereby mimicking the effects of cisplatin. Consequently, the virus would enjoy partial *MSH3* silencing, which could be enhanced further by engineered anti-*MSH3* siRNAs used as anticancer agents. This proposition stems from the central hypothesis of this article—the multifunctionality conferred by the Ambati-type sequence homology. While entirely speculative, these considerations could inform studies on SARS-CoV-2’s impact on DNA damage repair pathways, tumorigenesis, and responses to cancer treatments. Created in BioRender. Mueller, S. (2026) https://BioRender.com/s8grvb4.

In all, the findings by Zhang and El-Deiry [43] may indicate that suppression of p53 is a viral countermeasure to support its propagation, potentiated by agents such as cisplatin, possibly through dysregulation of host DNA repair. Viruses that can express siRNAs targeting MSH3 are perfectly aligned with this proposition, as they could be reproducing cisplatin-like effects. In turn, the ensuing DBS-repair inhibition likely places them under pressure to evade p53-driven cell death. This hypothesis, summarized in Figure 6, may support future studies on viral impacts on tumor biology.

The above raises the concern that viruses that harbor Ambati et al.-type sequence homologies involving the reverse complement to an *MSH3* portion could, thereby, target *MSH3* which would cause numerous detrimental effects, particularly in a cancer context:

- Generally, *MSH3* suppression results in unrepaired damage and mutations, which, in turn, activate oncogenes or inactivate tumor suppressor genes that ultimately cause genomic instability. This increases the risk of cancer [74]. Thus, viruses that harbor potential MSH3-siRNAs may, likewise, promote cellular transformation by co-opting host HR and the fidelity of DSB repair.
- The virus-induced inhibition of p53, even when exposed to anticancer drugs, severely hampers cancer patients undergoing chemotherapy.
- The host–pathogen interplay in such a situation is insufficiently understood for CoVs. The full scope of downstream effects triggered by *MSH3* silencing, and how this could drive viral evolution, is unknown.
- Besides biosafety concerns involving accidental and unrecognized recombination events with RNAi potentials, they may also lend themselves to malicious exploitation.

## 6. Implications for Biodefense Preparedness and Response

The above scenarios were only described in abstract terms, mandating their validation under adequately high-scaled laboratory conditions. For example, knockdown experiments involving different viral/siRNA co-transfections may demonstrate a variable degree of recombination events. Additionally, specific lab-imposed pressure could show which recombinants, if any, would be selected for. Such data could reveal unappreciated processes that could foster CoV evolution. Aside from potentially enabling unintended laboratory exposure to such more dangerous recombinant viruses, this could also harbor potential for deliberate misuse.

This section examines the various postulated scenarios to distill where the key vulnerabilities reside, and, based on this insight, aims to strengthen existing biorisk regulations, as suggested below.

### 6.1. The Potential of Bioweapons Research Masquerading as a Beneficial One

Several scenarios are described in [8] of how some of the underlying features that support Ambati et al.-like recombination events could mask research done for nefarious purposes. Declared as cancer research or other experiments with seemingly benign agendas, the recombination of synthetic mRNAs with those of certain viruses could make them more dangerous without being readily recognized or even suspected.

### 6.2. Bypassing Traditional and Advanced Biorisk Management Regimes

Traditionally, one of the most serious aspects of pathogen research was the recognition of overlapping mutations to confer malicious functions, such as host range expansion and increased virulence. Specifically, mutations in the amino acid sequence of the surface protein of several viruses can have a significant impact on viral function and immune recognition. For example, a single mutation—E1-A226V—in the chikungunya virus, which is normally spread by *Aedes mosquitoes,* alters the virus’s vector specificity and dramatically boosts its epidemic potential by markedly increasing infectivity in *Ae. albopictus.* Similarly, the Ebola virus (EBOV) glycoprotein (GP) mutation A82V was found to be the major contributing factor in its increased infectivity, pathogenicity, and fatality [76]. The most well known situation which triggered fears of covert biological weapons development likely involves H5N1, where a single mutation in the HA gene enables preferential binding to human-type receptors, enabling cross-species transmission and airborne spread in mammalian models [77].

Such characteristics create opportunities for misuse when pathogens are introduced into susceptible environments, where harmful mutations may arise inadvertently or through misguided or deliberately misrepresented laboratory experimentation. Those situations served as the impetus to tighten DURC policies and enforce a stricter review of the associated agents [78,79,80].

The concerns described above depict a related bioweapons threat scenario that, while overlapping with DURC characteristics, has unique aspects that may foster unrecognized attack potentials. This is not to say that the model envisioned implicates Moderna or the patent identified by Ambati et al., nor that SARS-CoV-2 is a bioweapon. Yet, the feasibility of the scenario illustrates the imminent potential for analogous malicious exploitations. Overall, the novel gaps rest on the following key factors:

- Sequence multifunctionality: One of the main points made in [8] and extended above is that the potential integration event of the FCS in a susceptible CoV might not emerge because of some evolutionary advances via this cleavage site per se, but rather, in the context of nuclear trafficking. Instead, the direct consequence would be the relocation of the spike protein/mRNA into the nucleus, conferring some advantage as commonly exploited by nuclear CoVs. In return, the overlapping function as FCS would additionally enhance viral entry and contribute to the unique pathogenic features of the virus.
- The sequence insert may involve the reverse complement of a synthetic sequence rather than one with a targeted activity.
- Besides the FCS/NLS overlap, the one between the FCS and the reverse of the *MSH3* sequence portion establishes another multifunctionality aspect.
- These traits, applied in these ways, result in a combination of the individual effects (transitivity).
- The involvement of the well documented *MSH3* gene and/or a patented sequence information would hardly trigger biorisk scrutiny and oversight, as it would be associated with benign and harmless research.

These considerations place considerably less emphasis on a potential “pathogen of concern” and shift it to “sequences of concern” (SoCs), in line with a recently developed improved policy for biorisk research involving microbial modifications [9]. Over the course of several years, this project—designed to fill the many gaps in existing policies and practices—places SoCs at the heart of biorisk management. The vast framework developed specifically also highlights multifunctional SoCs as more concerning than those with a single function and details numerous SoCs with multiple functions from bacterial, viral, and eukaryotic pathogens.

Even with the highly detailed, carefully tiered definition and categorization of SoCs, the scenarios described above would nevertheless evade this advanced and rigorous biorisk regime.

### 6.3. Subverting the SoC Framework and Opportunities for Improvement

Research that could lead to the integration of the FCS would likely not be identified even by the SoC policy [9], as it would evade some of the foundational aspects considered therein.

The SoC framework hinges on a very concrete conception of SoCs and their hierarchical ordering in terms of their potential to cause harm. The resulting metric is underpinned by an extensive, detailed analysis of annotated sequences and the critical functions of SoCs, supported by Machine Learning pipelines and advanced bioinformatic software. This analysis suggests that SoCs are not only most abundant in viral genomes but also that the great majority of the encoded sequences of nonviral microbes play no role in pathogenesis. As such, this conception would have missed key aspects relevant in the theory above, raising the question about additional concerns:

- One may not have to begin with a clearly defined SoC, and, nonetheless, end up with such. Specifically, the above does not start with a defined pathogenic sequence per se. Instead, it hinges on a synthetic fragment associated with a human gene. Thus, a SoC-guided biorisk management approach would not flag *MSH3* as a concerning sequence. Indeed, before the work by Ambati et al. [1], there was no reason to do so. More generally, however, the SoC approach, including its rubric, may not be easily extendable, if at all, to synthetic sequences, and particularly, as these are often proprietary.
- The comprehension of what functions are concerning is necessarily limited. Specifically, even though numerous immune-subverting activities related to SARS-CoV-2 SoCs are listed in [9], the key players analyzed above are not covered. Other functions, such as “within-cell motility,” are regarded as of “lowest concern,” and it does not seem that this category includes functions like nuclear localization. Also, the hijacking of the DDR mechanisms is not mentioned, and the possibility that some viruses subvert these by activating them seems to contradict the hierarchy that “immune-subverting” sequences would be “the worst” of SoCs. Even though the list of functions of SoCs presented in [9] is extensive and their key aspects come across as compelling, such omissions or seeming counter-examples merely reflect the very often very irrational and seemingly illogical pathogen–host interactions and astonishing ways in which certain viruses escape host immune recognition and antiviral defense processes, and our limited comprehension of the complex host–pathogen interplay.
- Besides, or rather than, a sequence of interest itself, it may be its reverse complement that has a harmful property. Multifunctionality further vastly extends the scope of concerning candidates.
- The harmful attributes may not be caused by specific features of a SoC, or even some of its multiple functions, considered separately. The combined multifunctionality of these elements can produce synergistic effects that are not apparent when each function is considered in isolation.

### 6.4. Potentials of Sequence Multifunctionality to Be Diverted for Malicious Use

Overlapping or double-function genetic elements are shockingly common in natural proteins and synthetic biology. Many of these are listed in [9]. As indicated, this work focuses on SoCs as characterized by harmful effects, such as cytotoxicity, tissue degradation, organ impairment, inflammation, and immune-evasion mechanisms (e.g., suppressing immune signaling, resisting phagocytosis), alongside properties that facilitate spread, adhesion, invasion, and similar processes. According to the conception of sequences “of concern,” even those listed with multiple functions constitute functions with “concerning” attributes. However, it is possible that perilous engineering may exploit additional aspects of genome multifunctionality. The following subsection gives a few illustrative examples.

#### 6.4.1. Multiple Functionalities Could Conceal a Hidden Malignant Function When Not Recognized

The following examples demonstrate natural occurrences comparable to the situation above, where the NLS/FCS functionality could be hidden within the seemingly innocuous contexts of *MSH3* gene and cancer research.

- Similar or same proteins playing opposing roles: Plants respond to pathogen exposure by activating the expression of a group of pathogenesis-related (PR) defense proteins. Surprisingly, copies of genes encoding PR-like proteins are also frequently identified in the genomes of fungi and other phytopathogens, which employ these proteins to bolster their virulence and suppress plant immunity. The surprising fact that emerges is that these conserved proteins act as antimicrobial agents when produced by the host plant but simultaneously suppress plant immunity when generated by the pathogen [81]. These multifunctional proteins—used by plants as antimicrobial agents yet co-opted by pathogens to enhance virulence and suppress host immunity—are only now beginning to be understood. Presenting only one facet of the protein’s activity while masking the other creates an opening for malicious use in sensitive contexts, which may, for example, foster pathogen takeover of susceptible plants. Analogous, more general dual functionalities of similar proteins in animals and humans could have unprecedented applications as biological warfare agents, for the malicious effects could only be triggered in specific contexts.
- The HIV-1 Rev protein’s arginine-rich motif (ARM) is both an RNA-binding domain (RBD) and an NLS: As indicated above, an NLS may not be on the top SoC list. Although the framework does not mention it directly, an NLS would likely fall into the lower-risk category of SoCs under Godbold et al. [9], given that intracellular protein trafficking is deemed one of the least problematic functions. Likewise, the RNA-binding capacity does not seem to have triggered their classification as a function related to a SoC, for these are primarily considered in the context of pathogenic functions. As a result, this double functionality in the HIV-1 Rev protein might escape oversight. Nonetheless, it harbors a concerning attribute, which is to facilitate the nuclear export of viral mRNAs to the cytoplasm, where they are either translated or packaged into assembling virions [82,83]. Therefore, even though critical for HIV-1 replication, the concerning feature of the HIV Rev motif could be concealed behind either or both of the above functions, which appear benign.
- Engineered fusion proteins often contain sequences designed to perform multiple roles, e.g., involving signal peptides that direct proteins to particular destinations in the cell while incorporating protease cleavage sites that allow the protein to become active or to be released from a membrane-bound state [84]. This dual functionality, targeting via the signal peptide and subsequent activation via proteolytic cleavage, is a common strategy for protein therapeutics [85]. Fusion proteins that contain concerning sequences might nonetheless escape SoC oversight and regulation when they (a) are expressed in non-replicating, nonpathogenic, or cell-free systems; (b) contain benign domains that mask a covert, malicious role; and (c) combine functional domains or underappreciated multifunctionalities in novel ways whose attributes and impact may not be foreseen or well characterized.

#### 6.4.2. Existing Biorisk Comprehension of Overlapping or Multifunctional Elements Requires Substantial Revision

Various biosafety and biosecurity initiatives focus on stabilizing engineered organisms and preventing their escape or horizontal gene transfer. They employ multilayered biocontainment that integrates traditional physical/administrative controls with genetic design. These approaches also target genetically overlapping or multifunctional elements.

For overlapping gene constructs, it is tempting to think that such structures might increase evolutionary constraint. This rationale underpins biorisk considerations of synthetic constructs based on the following considerations [86].

- Synthetic constructs with overlapping genes are thought to stabilize synthetic designs because mutations impact both genes simultaneously. Such a gene stabilization is aimed to reducing horizontal gene transfer and guaranteeing biocontainment.
- It is thought that gene overlaps may help prevent the unintended dissemination of genetically engineered DNA, in that artificially created overlaps enhance the evolutionary stability of engineered genes by embedding them within an essential gene (such as one conferring antibiotic resistance).
- Specifically, Leonard et al. [86] created overlapping genes by insertion of an “inner” gene, encoded in an alternate frame, into a flexible region of an “outer” gene. By directly linking the evolutionary fate of the engineered gene (the inner gene) with that of an overlapped gene, this coupling is believed to reduce the likelihood of mutations and stabilize the engineered gene. This is because mutations disrupting the inner gene would also impair the outer gene’s function, which is critical for the organism’s survival (e.g., antibiotic resistance).
- In a concrete demonstration of this, Leonard et al. [86] created a new overlapping gene pair of bacterial toxins within an antibiotic resistance gene. Selecting bacterial toxin genes as the “inner genes” is intended to limit horizontal gene transfer (HGT) of the resistance gene, thereby reducing the risk of spreading antibiotic resistance.
- The rationale for this is that any transfer of the resistance gene to a new host would also transfer the toxin due to their overlap. If the recipient organism lacks the matching antitoxin, toxin expression becomes lethal, blocking successful transfer and thereby limiting HGT.

This method aims to deliberately stabilize an engineered gene by directly linking its evolutionary fate with that of an overlapping gene. However, from a biorisk perspective, this premise raises serious questions. The host used by Leonard et al. was *Escherichia coli.* In an adversarial context and involving various human microbial strains, it seems possible that the above could be transformed into a covert bioweapon, such as when used under the guise of a therapeutic. First, when the transmission of the toxin gene is allowed or even fostered, this could wipe out substantial proportions of microbial communities, which could have devastating effects on human health.

Additionally, the situation envisioned by Ambati et al. [1] suggests some potential overlaps that challenge the presumed safety of the Leonard et al. [86] method, making it susceptible to misuse and potentially enabling the dissemination of covert biological weapons.

- Biocontainment by the Leonard et al. method hinges on the survival of the host. The choice of an antibiotic resistance gene as the outer gene is expected to ensure that mutations disrupting the inner gene would likely impair antibiotic resistance, leading to the host’s death under selective pressure.
- This rationale is similar to considerations that challenge the feasibility of an Ambati-type et al. postulate. In such a case, selective pressure on an RNA virus, e.g., to hinder its nuclear import/export, might be expected to harm RNA viruses. However, escape mutants would gain enhanced capabilities, allowing them to evade host antiviral defenses whilst hijacking DDR responses.
- Likewise, then, it seems possible that, as the evolutionary fate of the overlapping genes is tied together, selective pressure could enable the development of novel bacterial escape mutants that would make them even more resistant. This could facilitate the spread of resistance to multiple antibiotics and essential drugs, and enable the covert use of biological weapons against patients with bacterial infections under the guise of a therapeutic intervention.

In sum, the above considerations, inspired by the Ambati et al. hypothesis, suggest that the concept of overlapping or multiple-function genetic elements in an adversarial context has not been adequately examined. While the framework proposed by Godbold and colleagues [9] represents a major advance for biosafety, protecting against the intentional exploitation of related vulnerabilities remains a daunting challenge. Our incomplete comprehension could potentially enable the design of novel biological weapons involving bacteria or viruses prone to form escape mutants under selective pressure, albeit often in ways that seem irrational. Both DNA and RNA viruses exemplify how pathogens can co-opt host immune defenses in ways that initially appear counterintuitive. Such difficult-to-understand features are ripe for malicious exploitation. When attention is focused on a key target gene, an overlapping gene can conceal unexpected or harmful activity, camouflaging its unexpected or malicious behavior.

### 6.5. siRNAs Engendering Undefined Activities vs. Unintended Integration of De Facto siRNAs

Unintentional risks and potentials for deliberate subversion involving short synthetic RNAs may arise in both directions. In one case, siRNAs may be utilized on purpose, e.g., during in vitro transfection, but then acquire additional functions when unintentionally integrated into a viral genome. Conversely, synthetic RNAs of variable lengths, if acquired by viruses through recombination, may be processed as short RNAs that are functionally equivalent to siRNAs with unknown targets.

**Figure 7:**
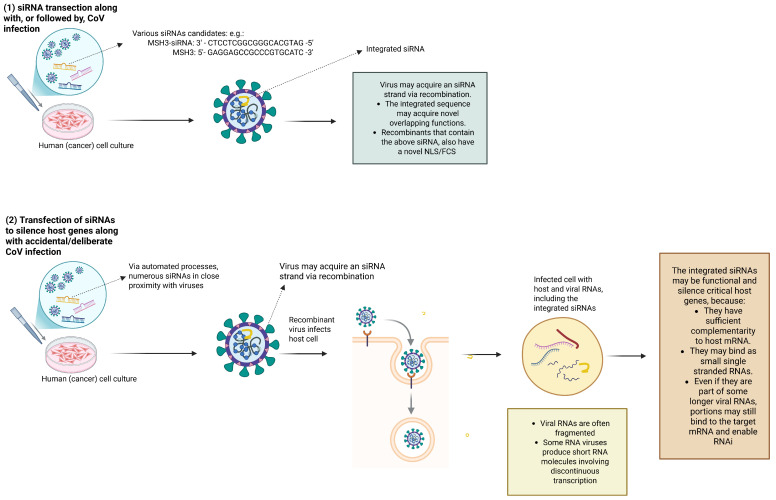
The integration of short-interfering RNAs (siRNAs) envisioned above may evoke biorisk concerns in two different ways: (1) In vitro siRNA/viral co-transfection could, as described, result in the integration of sequences into the viral genome with entirely unintended biological activities. Some of these may have overlapping functions. (2) Deliberate siRNA screening, e.g., to silence key host genes, when unintentionally acquired by RNA viruses, may be expressed as such functional siRNAs akin to in vivo RNAi systems employing chimeric viruses. Created in BioRender. Mueller, S. (2026) https://BioRender.com/yvq0her.

The two scenarios of siRNAs unintentionally involved in viral recombination, as envisioned above, are depicted in Figure 7.

#### 6.5.1. From Deliberate Gene Silencing to Unintended Biological Functions: An Underappreciated Biorisk

One of the main concerns above was that the antisense sequence of some siRNA could have an unrecognized biological function (Figure 4). That is, even if the original siRNAs are well defined, their complements can also play potent roles in viral evolution when expressed. For instance, an siRNA candidate intended to target the human *MSH3* gene may involve an FCS in a CoV. The induction of such unintended functional overlaps is not a unique occurrence.

The potential of the reverse complement sequences of siRNAs/miRNAs overlapping with sequences with distinct functional roles is well documented in the literature, both in plants [87] and in animals. For example, already in 2008, Stark et al. [88] found that Drosophila iab-4 contains miRNAs involving both sense and antisense strands. They further confirmed that both miRNAs are expressed throughout fly development and induce different phenotypes when ectopically expressed. The authors believe that such sense/antisense miRNAs could not only restrict each other’s transcription but also target distinct sets of genes. They also provided evidence that sense/antisense miRNAs are much more generally employed in different contexts and species.

Therefore, if a research project involves the silencing of specific genes to target a single function, unintended regulatory effects may arise, particularly since many viruses utilize both their sense and antisense sequences during their life cycle. Considering that these mechanisms are insufficiently understood and addressed in biorisk analyses, they may be particularly prone to malicious exploitation.

#### 6.5.2. Unintended or Covert Integration of Sequences Which, When Expressed by a Virus, Function as siRNAs

Above, the concern that viruses may acquire synthetic RNA sequences and gain unexpected RNAi capacities was inspired by the work of Chambers et al. [46]. In their experiments, these authors deliberately evoked RNAi by adding a synthetic RNA into the influenza virus genome. The engineering of such chimeric viruses for in vivo RNAi is an established technique [69]. It is based on the naturally occurring RNAi mechanism that enables the silencing of specific genes by degrading mRNA before it can be translated into protein. However, it does not seem that the analogous processes fostered by CoV recombination have been investigated. Many foundational aspects of the potentials and features of such recombination-based RNAi processes remain to be elucidated.

There is, more broadly, no complete understanding of the fundamental characteristics of siRNAs, as illustrated, for example, by the United States patent application US 18/566,561 [89], which employs much shorter constructs (from 15 nucleotides upwards) than the 21–24 nucleotides often regarded as necessary. During the classical process, siRNA duplexes are incorporated into the RNA-induced silencing complex (RISC), where the antisense (guide) strand is retained and used to identify complementary mRNA sequences. The passenger strand is thought to be degraded. The above describes the concern that cell culture experiments could similarly integrate small RNAs into RNA viruses, enabling them to bind to new targets. In this case, the orientation is arbitrary, and short RNAs in either direction could play such a role, especially as viruses often rely on intermediate templates. CoVs, in particular, do not replicate their genome consecutively from the beginning to the end but via a convoluted process involving subgenomic RNAs. This piecemeal processing could further increase the availability of short RNAs for unanticipated binding to, and reduction of, various host mRNA molecules and of the corresponding proteins.

Functionally, the RNAi silencing reaction itself does not involve the dsRNA precursors and is mediated by only one strand, which specifically recognizes and binds the mRNA target. In this manner, RNA viruses, when they recombine with and express synthetic RNAs, may directly acquire RNAi function resembling mature si/miRNAs, thereby bypassing the classical dsRNA precursors required for classical siRNA silencing.

It also seems possible that virally expressed single-stranded RNAs with unexpected characteristics could engender RNAi. For instance, supported by structural features, it may be that the virally expressed recombined siRNAs are substantially longer than 24 nucleotides and still bind to portions of host mRNAs. This notion is consistent with the observation that RNAi can also be mediated by small non-coding RNAs that act directly as guide sequences and require only partial complementarity to bind target mRNAs, typically within their 3′ untranslated regions (3′ UTRs) [90].

Commercially available systems and therapeutic initiatives aimed at evoking RNAi usually mediate only transient silencing because their concentrations in the cytoplasm are diluted over time with successive cell divisions [90]. Yet, when integrated into a viral genome, such RNAs could maintain their activity throughout the viral life cycle. This seems particularly concerning in an environment of impaired immune responses and prolonged presence of the viral RNAs.

### 6.6. Recommendations

The sequence that encompasses the SARS-CoV-2 FCS is generally scrutinized in relation to the origin of this virus. On the other hand, this study, set apart from origin arguments, exposes feasible processes and mechanisms to promote CoV recombination. It is unclear to what extent these underpinnings may have been grasped if the Ambati et al. scenario were exclusively examined through the lens of the virus’s origins. Those intending harm may not care about the past and are more likely interested in maliciously exploiting unappreciated vulnerabilities.

The analysis of the postulated event, investigated from a purely theoretical perspective, has exposed numerous aspects that can inform biorisk policy and oversight.

- The prediction of the bioweapons potential via the agent per se, e.g., based on their potential to cause harm, is very restricted. This is the pivotal insight of the SoC framework developed by Godbold and colleagues [9]. This potent approach may be strengthened by incorporating additional sequences and functions of concern, as exemplified above. In addition, the model could be enhanced by accounting for the effects of the reverse complement, not just the individual sequences, and by exploring possible synergistic or overlapping activities.
- Even though predicting transitive and synergistic outcomes may be challenging to model, the SARS-CoV-2 NLS/FCS overlap demonstrates the feasibility and biological relevance of such effects.
- Generally, it is expected that vulnerabilities and hazardous scenarios derive from gain-of-function studies. Ironically, the above-mentioned concerns arise from loss-of-function experiments instead.
- Biorisk concerns may emerge in the context of gene silencing, particularly when this involves a library of short RNAs with (potential) regulatory function. Regulatory RNAs, when targeting viral genes, can directly put selective pressure on viruses. Additionally, when transfected into cell culture, synthetic RNAs targeting host genes or other short RNAs deemed harmless can also be exposed to the virus, for example, to examine cell survival. When viruses acquire siRNAs intended to silence host genes during cell culture experiments, this raises the concern that they could be expressed, inducing RNAi that suppresses key host processes. Whereas the situation of bacteria integrating antibiotic resistance genes is a well studied problem, the analogous situation involving viruses does not seem to have been appreciated for its biorisk potential. The implications could be profound.
- siRNAs targeting *MSH3* are also widely used for chemotherapy. For example, the United States patent application US 18/566,561 [89] describes the characteristics of the siRNAs based on dsRNAs involving a sense or antisense strand which “is complementary to 19 contiguous nucleotides of an MSH3 gene.” Inhibition or knockdown of *MSH3* via transfection of siRNA duplexes is demonstrated using a cell-based assay and involves a large library of sense/antisense pairs to target *MSH3*. However, the concern of recombination with a CoV, including when these are present as contaminants, does not seem to have been described.
- Practically, appropriate siRNAs may not a priori be clearly defined. In an automated setting that involves large siRNA libraries, this may include candidates that may not have the intended silencing capacity. The approach of experimentally validating and identifying those candidates with optimized function would inherently involve the transfection of countless short RNAs into sensitive cell environments.
- A library of potential candidates that, for experimental validation, also harbors the concern for recombination events with viruses not intentionally analyzed but accidentally left over in the culture as contaminants. The converse is also true. Extensive gene-silencing work may produce stray short RNAs that remain undetected, thereby risking accidental exposure in other pathogen-related studies.

This process of gene silencing using a massive library of synthetic siRNA candidates appears harmless. Yet, in the wrong hands, it could obfuscate the integration of an NLS/FCS or related pathogenic features. Disturbingly, these and the other features identified above resemble the perfect scenario for clandestine biological weapons development:

- The entertainment could be disguised as benign, e.g., masquerading as cancer research.
- The agents involved can be portrayed as harmless, including non-human CoVs unable to infect humans and well described entities, such as short gene sequences resembling human genes.
- Harmful siRNAs that may potentially be acquired by CoVs could be hidden inside an extensive library, thwarting any practical manual screening for dangerous activity.
- The above focuses on one hypothetical scenario, involving the sequence encompassing the FCS, of how experimental conditions and viral evolution could converge and create biorisk hazards. It is unlikely this is the only such potentially perilous integration scenario. Other sequences, processes, and circumstances similar to the above, able to cause unrelated detrimental recombination events, cannot be ruled out.

The sobering prospect of this is that various laboratories engaging in related research experiments could be infiltrated to secretly create a novel, potent biological weapon able to infect humans or specifically targeted animals. Today’s unprecedented technological throughput, exemplified by the ability to conduct extensive gene-silencing screens that could be secretly weaponized, puts synthetic biology at a critical juncture. The enormous quantity of siRNAs, cloaked within a seemingly harmless experiment, would render detection of hidden dangers practically unfeasible for oversight agencies.

Because true identities of individual agents and processes can be camouflaged, obscured, or switched, it now appears that the involvement of humans, including their wisdom and unique capacities, is more imperative than ever. Whereas numerous laboratories could be hijacked, I firmly believe that the vast, overwhelming majority of researchers in this field are very conscientious and honest. In sum, this human factor may prove critical, as emphasized by other disciplines that could be maliciously exploited (Table 3).

The inherent gap [7,93] between a technological depiction of harmful biological agents and players can obfuscate covert biological weapons development, making the identification of malicious components extremely challenging. It perfectly parallels the biblical episode, including the fascinating solution attributed to King Solomon’s wisdom. Analogously, it is suggested that at this critical stage of synthetic biology, biosecurity can most effectively be supported by the expertise, wisdom, intuition, and insight of every individual lab worker involved (Table 4).

## 7. Limitations and Synopsis of Future Research

As with any entirely theoretical work, the limitation of the above is that it lacks experimental validation. However, the individual aspects described are all testable in the framework of current knowledge. The sequence homology noted by Ambati et. al. is not merely a bizarre feature from a scientific perspective. By its very existence, it encompasses overlapping functions and biological consequences that are poorly understood. Whereas several feasible consequences were envisioned above, based on analogous scenarios involving lab research and CoV biology, they have been merely theoretically described and await validation in high-scale laboratories.

All the envisioned scenarios are based on direct parallels between a known laboratory technique or biological mechanism. They are presented to demonstrate how recombination, possibly fostered by certain laboratory conditions, might arise and produce unforeseen biological outcomes through sequence overlaps, without implying that such an event has occurred.

The above also did not focus on the biological and clinical relevance of the sequence overlap specifically related to SARS-CoV-2. Rather, it indicated the feasibility of such types of recombination events and the unrecognized sequelae, including CoV evolution and unrecognized aspects of the pathogen–host interrelationship.

This work was motivated by the recognition that, without sufficient awareness, related types of experiments could result in similar events of unappreciated biological relevance. The types of postulated recombination events, which may unwittingly endow pathogens with unrecognized overlapping features, survival benefits, and increased pathogenicity, could result in the unintentional exposure to or release, thereby endangering laboratory workers, the public, and the environment. However, covert recombination events involving harmful sequences with unexpected multiple functions could potentially also be leveraged for harmful intent. This duality is a known dilemma with all security disciplines. On the one hand, pointing to a novel gap could inform mitigation and response measures. Conversely, publicizing such data could also aid those intending to misuse this information. Since the introduction of DURC research and the recognition of these fundamentally opposing issues, the synthetic biology community has struggled to determine the right balance. The above investigation has, therefore, provided a comprehensive analysis of the situation without ascertaining which of the individual aspects are more or less susceptible to either unwitting viral evolution or malicious exploitation. Overall, it aimed to merely depict the feasibility of the scenarios in their broad generality. It also does not attempt to describe completely new gene inserts that have not been discussed in the literature—information that could aid nefarious activities.

The sequence homology reported by Ambati et al. has not received the biorisk scrutiny it warrants, and ignoring it will not diminish the risk. Doing so will not make these sequences go away. Nonetheless, it is unclear which of the above aspects inspired by this extraordinary feature will prove most relevant in reality, and some of the vulnerabilities may require specific contexts, such as a compromised immune microenvironment.

Despite its abstract nature, the above considerations extrapolate from established knowledge, albeit with a forward-looking orientation to help mitigate any biorisk implications. The comprehensive analysis has proposed several novel mechanisms and future research directions. Table 5 summarizes the mechanistic analogies, scenarios that call for greater experimental caution, and specific translational insights.

## 8. Conclusions

The Ambati et al. [1] postulate concerning the integration of a synthetic sequence encompassing the SARS-CoV-2 FCS is usually interpreted to potentially imply a link to the laboratory genesis of this virus. In turn, the suggested recombination event between a synthetic RNA and any suitable CoV has contributed to hefty debates about the evidence, or lack thereof, of the viral origin. Yet, this particular sequence cannot suggest any information about the actual viral backbone, which is profoundly necessary for the origin question. On the other hand, such types of recombination events are of high interest in and of themselves.

**Figure 8:**
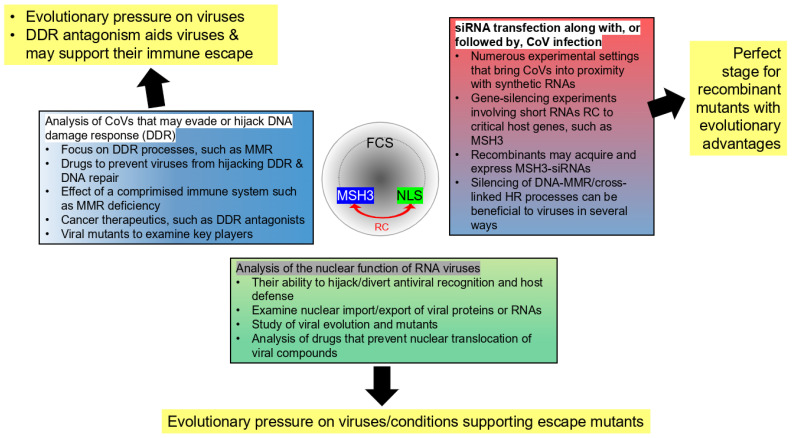
Here, the acquisition of a sequence surrounding the SARS-CoV-2 FCS and that of analogous recombination events is analyzed for their feasibility and ensuing biorisks. The analysis rests on plausible laboratory conditions that could facilitate recombination with a short synthetic RNA sequence. Rather than scrutinizing the viral origin, which is distinct from the FCS per se, it investigates unappreciated mechanisms that may emerge in the context of cancer research. Key orientations and types of research experiments conducive to this integration are depicted in the red/blue/green-shaded rectangles. The colors are intended to match the key drivers of these processes, as shown in the center, involving genetic multifunctionalities, where a single gene is involved in multiple cellular processes or biological functions. Whereas the FCS has garnered substantial attention, the overlapping NLS is well confirmed but has received comparatively little attention. Beyond this, the sequence homology with the reverse complement (RC) of *MSH3* may enable yet another function via the expression of small-interfering RNAs (siRNAs) that target key host DNA repair processes. The outer parts of the figure highlight the evolutionary pressure or survival benefits created by some of the experiments, which could support the covert development of escape mutants with improved evolutionary advantages, supporting the survival of some recombinant mutants. That these scenarios are not entirely speculative is evidenced by known RNA virus traits and analogous in vivo gene-silencing experiments via engineered viruses. Specifically, in laboratory experiments with chimeric influenza viruses, an integrated sequence complementary to MSH6 was processed into siRNAs, which silenced essential host pathways. Facilitated by RNA virus recombination traits, this suggests that analogous recombinant mutants equipped with the overlapping three functional elements could acquire related evolutionary benefits. Such extensive gene knockdown experiments could explain why a potential CoV recombination event could comprise a very short synthetic RNA sequence and achieve unintended biological functions, which has received little scrutiny from a biorisk perspective.

Instead of considering past events, here, the perfect match between the sequence encompassing the SARS-CoV-2 FCS and the reverse of an *MSH3* sequence portion was used as a springboard to suggest unrecognized biological mechanisms, host–pathogen interactions, and novel biorisk concerns. Here, the analysis of potential laboratory experiments and their relationship to viral evolution in a broad context, separate from the origin question, highlighted unappreciated bioweapons development potentials. Extending prior work, additional testable scenarios were described that support the feasibility of an FCS/NLS recombination (summarized in Figure 8). They are no proof of the origin of this detrimental feature of SARS-CoV-2, but they highlight the imminence of research contexts that may result in types of research that could endanger biosafety or even be harnessed for nefarious purposes without being readily detected. Indeed, CoVs are predisposed to the types of recombination events described. They tend to exploit various nuclear features and hijack DNA damage response and repair processes. Because these traits are poorly understood, they may aid covert bioweapons development and complicate attribution, especially since such features are unexpected in RNA viruses.

A range of circumstances in which cancer research activities may inadvertently exert evolutionary pressure on the virus has been identified, potentially supporting harmful viral recombination. The troubling aspects would likely escape biorisk surveillance and oversight, including the new USG DURC-PEPP Policy and prior policies (https://ipo.rutgers.edu/rehs/bio-dual-use, https://research-compliance.umich.edu/research-safety/durc-pepp-policy, https://www.ehs.washington.edu/biological/biological-research-approval/durc-pepp, last accessed on 17 January 2026), where the danger of the experimental outcomes or actions must be “reasonably anticipated.” The more rigorous SoC framework [9] also overlooks key concerns outlined above, including the indistinguishability of some sequences and their functions, which may prevent them from being recognized as hazardous. Indeed, sequences with multiple functions can easily disguise malicious attributes, especially when combined (synergistic/transitive effects), in a context where one function can come across as benign, and/or, as was documented above, as many features of how viruses escape host immune recognition and defense are incompletely understood, illogical, or entirely unknown.

In this context, the recombination between CoVs and short RNAs is particularly worrisome as it could camouflage RNAs with regulatory capacities. The acquisition of sequences (reverse) complementary to host mRNA could downregulate critical host gene functions or have other unintended functions during the pathogen–host interplay. Whereas the article focused on potential future events biorisks informed by the Ambati et al. sequence homology in SARS-CoV-2, this also prompted questions of how such a sequence overlap could drive future viral evolution. Moreover, a deeper understanding of the underlying sequence multifunctionality is crucial for anticipating downstream effects, including those relevant to vaccines that employ the same genetic information.

In a context of laboratory experiments that include (i) cancer-focused manipulation of DNA repair pathways, (ii) selective pressure from DDR inhibition, (iii) drug-induced inhibition of nuclear transport, and (iv) reliance on siRNA libraries for the gene knockout of critical host genes, these factors, when examined in the context of oncogenic or oncolytic viruses, or CoVs as experimental tools or contaminants, could theoretically foster unaccounted recombination events in some CoVs. These could include multiple functionalities, such as NLS/FCS overlap or host gene-silencing functions, which could covertly enhance the infectivity and pathogenicity of the virus.

The last few decades have drastically enlarged technological throughput, for example, to create extensive libraries of short/regulatory RNAs to silence viral proteins or facilitate the knockdown of relevant host genes. Many such notable efforts are aimed at developing interventions to help defend against viral infections and serious disease outcomes. In very concrete research contexts, as detailed above, the inherent propensity of CoVs for recombination could foster the integration of an MSH3-siRNA, analogously to in vivo RNAi techniques, and endow the virus with substantial survival benefits. Thanks to the sequence homology, such a process could facilitate the integration of an FCS/NLS into various CoVs.

Faced with a seemingly irresolvable situation in synthetic biology, where the DURC dilemma has reached a breaking point, one may wonder whether technology will ever be able to turn the situation around. Interestingly, King Solomon did not proceed to resolve the identity of the living infant but evoked a response at a different level altogether. Likewise, it is hoped that decoupling FCS/NLS analysis from the issue of viral origin will help prevent potential misuse of the associated information, now that these vulnerabilities are brought to light.

## Figures and Tables

**Table 2:** Examples of experiments that combine MSH3 and viruses in cancer research.

Description	Comment	Related Experiments
Transfect a CoV into a cell line that over-expresses *MSH3*.	Overexpression is known to compromise DNA mismatch-repair mechanisms, thereby generating a dMMR condition similar to that seen in various cancers.	The consequences of CoV infection for individuals with cancer, notably SARS-CoV-2, have been widely studied (e.g., [39,40,41]
In this context, analyze the role of DNA damage repair pathways and test for potential inhibitors thereof.	Pharmacological inhibitors of DDR processes are extensively studied for cancer therapy. The precise link between this and CoV infection and evolution remains unclear.	The use of DDR antagonists on SARS-CoV-2 in the context of cancer has been suggested in [42].
In this context, or more generally, examine the nuclear involvement of CoVs, also in relation to nuclear import or export inhibitors.	Effective inhibitors could create substantial pressure on the virus. Conversely, certain escape mutants may have improved nuclear capabilities.	The effect of ivermectin, as an example of an importin inhibitor, has also been studied in relation to SARS-CoV-2 [42].
Thus, and also in a wider sense, analyse the properties of CoVs alongside other DDR components and the processes that link DNA damage to immune signaling.	The impact of CoVs on pathways of tumorigenesis and response to cancer therapeutics also influences host DNA damage sensing, response, and repair mechanisms. For example, a loss of p53 function is a known driver of cancer development and confers chemo-resistance [43].	Zhang and El-Deiry [43] specifically studied the effect of SARS-CoV-2 on p53 signaling in cancer cells.

**Table 3:** Framework for ensuring safety and addressing indistinguishability issues, informed by other disciplines.

Aims and Values	Rationale/Approach	Key Principles
Foster transparency and accountability	Transparency in processes and decision-making is a prerequisite for building trust and allowing for post hoc analysis.	The 2017 Asilomar AI principles (https://futureoflife.org/open-letter/ai-principles/, last accessed on 17 January 2026.): A culture of cooperation, trust, and transparency should be fostered among researchers and developers of AI, as a core principle. AI systems may cause harm. In such an event, it should be possible to ascertain why. Designers and builders of advanced AI systems are stakeholders in the moral implications of their use, misuse, and actions, and must assume responsibilities.
Ensure accountability and allocate liability in the case of harm	This is a core principle of the IEEE Global Initiative on Ethics of Autonomous and Intelligent Systems (https://apo.org.au/sites/default/files/resource-files/2017-12/apo-nid123376.pdf, last accessed on 17 January 2026). One central driver is the recognition that “The convergence of intelligent systems and robotics technologies has led to the development of systems with attributes that simulate those of human beings…” which requires ensuring accountability and allocating liability when such systems cause harm.	Legal requirements mandate transparency, participation, and accuracy, including: “Parties, their lawyers, and courts must have reasonable access to all data and information generated and used by such systems employed by governments and other state authorities” “The systems should generate audit trails recording the facts and law supporting decisions and they should be amenable to third-party verification” “The general public should know who is making or supporting ethical decisions of such systems through investment” The logic and rules embedded within the respective systems “must be available to overseers thereof, if possible, and subject to risk assessments and rigorous testing”
Foster traceability	Implement logging and auditing systems that are transparent, secure, and immutable.	Blockchain technologies. They are expected to advance healthcare via securing electronic health records; storing and sharing of records in a secure and privacy-preserved manner; ensuring tamper-proof, traceable, and non-repudiation-based mechanisms for health record and patient data; identifying counterfeit medicines; tracing and tracking of the medicine supply chain [91]. Explainable AI Techniques. Their main goal is to make the decision-making of complex models (“black boxes”) transparent and interpretable (https://www.ibm.com/think/topics/explainable-ai, last accessed on 17 January 2026). Note: These approaches implicitly assume a solution for dealing with proprietary data in case of dispute.
Encourage open documentation of methodologies, assumptions, and limitations	The limitations and assumptions of a system are often not properly documented.	Require a candid documentation of the system, including related data flows, performance, limitations, and risks (https://apo.org.au/sites/default/files/resource-files/2017-12/apo-nid123376.pdf, last accessed on 17 January 2026). “Criteria for such documentation could be: auditability, accessibility, meaningfulness, and readability.”
Adopt a range of strategies to quantify performance and risk, especially when new information becomes known	Include models that account for uncertainty and mechanisms for updating our beliefs about an event based on new data.	For example, Bayesian inference is a statistical method that uses Bayes’ theorem to update the probability of a hypothesis as more data becomes available. Unlike traditional inference, which relies on observed data, Bayesian inference is a statistical method that incorporates prior beliefs or existing knowledge, combines this with new evidence, and produces an updated belief (https://sustainabilitymethods.org/index.php/Bayesian_Inference, https://www.wolfram.com/language/introduction-machine-learning/bayesian-inference/, last accessed on 17 January 2026).
Leverage interdisciplinary ethical frameworks	Ethical frameworks grounded in first principles (e.g., harm minimization, fairness) can guide actions even in situations of ambiguity, technical undecidability, and when truth is obscured.	Engage diverse stakeholders (technologists, ethicists, policymakers). IEEE’s ‘ETHICALLY ALIGNED DESIGN’ principles are a set of foundational guidelines aimed at ensuring that autonomous and intelligent systems (A/IS) are designed and developed in alignment with human values and ethical imperatives. The Asilomar AI Principles require AI systems to be compatible with ideals of human dignity, rights, freedoms, and cultural diversity.
Encourage continuous monitoring and feedback	Ongoing observation can help detect emergent patterns that might reveal truth or risks.	Deploy automated real-time monitoring tools to track system behavior and flag anomalies. Use feedback from users or affected communities. Appreciate and foster the power of intuition, as e.g., seen with animal behavior or affected individuals, including in traumatic or stressful situations and in crisis management [92].
Cultivate epistemic humility, skepticism, and out-of-the-box thinking	Throughout history, the recognition of the limits of knowledge has often opened the doors of wisdom.	Foster research that questions assumptions and seeks disconfirming evidence. Foster a culture of humility and uncertainty. Popper’s Falsification Principle emphasizes that science progresses not by verifying theories through accumulating confirming evidence, but by rigorously testing and identifying conditions under which they are false (https://www.simplypsychology.org/karl-popper.html, last accessed on 17 January 2026). Support and train critical thinking, the core pillar of logical and mathematical sciences.

**Table 4:** Potential insights to be gleaned from the Old Testament parable about Solomon and the two women.

Insight Described from the OT Account	Potential Interpretation in the FCS Insertion Context
Once the link between the “baby” and “its mother” is severed, identifying the truth is much more difficult.	During a covert biological weapons program, individual components with biological activities can easily be disguised or swapped. In the language of the FCS/NLS insert: Even though the FCS/NLS sequence was first described for SARS-CoV-2, nefarious actors could take this information and insert it into other CoVs. Other sequences known to enhance the infectivity and pathogenicity of the virus may be transferred into a laboratory context with benign features, including CoVs unable to infect mammalians. Via digital information transfer, the hazardous sequence may be inserted into a different CoV in a remote laboratory in the context of experiments detailed above.
After the fact, and as truth identification was impossible at the level it first presented, a complete solution was possible by the wisdom of an unbiased observer, once he was made aware of the dispute.	If a hazardous sequence is infiltrated into a benign research environment, this would involve experienced and conscientious researchers. Once aware of the feasibility of unwanted recombinants, they could perform adequate monitoring strategies and oversight mechanisms.
King Solomon did not proceed to resolve the identity of the living infant. He did not perform any genetic tests, for example, to infer the real mother. Rather than addressing the dilemma at the level of true identity of the individuals involved, he evoked a response at a different level altogether. The term in 1 Kings translated from the Hebrew as “heart” could also be rendered (https://www.blueletterbible.org/lexicon/h7356/kjv/wlc/0-1/, last accessed on 17 January 2026) as “compassion,” “tender love,” “womb,” etc., and conveys the idea of a “gentle emotion of the mind.”	Regulations, oversight, and work environments that foster passion and compassion may also provide the framework for wisdom and deeper knowledge (intuition).
A “true mother” and a “wise king” have a boundless capacity for enlightened instinct, spontaneity, and commitment.	Analogous capacities of conscientious researchers may be overruled by automation and AI. They must be increasingly educated about the potential and risks of digitization and automation, and the new attack scenarios that may emerge. By creating an environment that emphasizes the value of each person involved, where individuals feel safe to express their true selves, including their innovative ideas and personal values, this may increasingly empower researchers to detect even those illicit developments that previously had not been thought possible. By protecting whistleblowers, encouraging authenticity and out-of-the-box thinking, individual researchers involved in synthetic biology may perfectly match the magnificent qualities portrayed in the biblical account.

**Table 5:** Taxonomy of the above postulated scenarios and “thought experiments” to describe novel biorisk concerns inspired by the Ambati et al. hypothesis and how these align with established knowledge.

Category	Suggested Scenario in This Analysis	Related/Analogous Known Scenario
Mechanistic analogies	Synthetic RNA-mediated recombination (Ambati-type insert): a short synthetic RNA (the 19 nt reverse complement of the proprietary *MSH3* sequence) could recombine with some CoVs during replication. This notion was first suggested by Ambati et al. and has remained unproven.	The envisioned mechanistic underpinning is analogous to natural recombination events commonly employed by CoVs and more clearly depicted in Figure 2.
	The integrated sequence into a CoV may be rather short.	Non-homologous recombination with CoVs takes place when the polymerase encounters a secondary structure or other hindrance during replication, or in the case of a short “acceptor” template.
	The FCS/NLS dual function may assist CoV evolution to enhance their nuclear presence.	This aligns with numerous examples of how CoVs, including SARS-CoV-2, utilize the nuclear translocation of their proteins/genetic material to their advantage.
	The involvement of the *MSH3* gene suggests some role of the host DNA damage response/DNA repair system that the virus uses for its benefit.	That RNA viruses suppress or subvert this host system is well described.
	CoVs may acquire RNA fragments that play a role beyond functional genes. Integrated sequences may be processed into virus-derived siRNAs and provide another layer of interaction with host RNA-silencing pathways.	The fact that viruses can express functional siRNAs to impact the host–pathogen interplay has been demonstrated for some viruses but is incompletely understood. Recombination events would further complicate our comprehension of their identify, function, and driving factors.
	The integration of an antisense *MSH3* sequence portion into a CoV or of other sequences reverse complement to host genes may endow recombinant mutants with the capacity to express these as siRNAs and downregulate critical genes and processes in the host genes.	The analogous scenario has been described during research with the influenza virus where de-facto siRNAs were intentinally integrated into its genome. *MSH3* gene inhibitors are emerging therapeutic agents that rely on siRNA duplexes where the antisense strand is complementary to at least 15 contiguous nucleobases of an *MSH3* gene.
Translational insights (1)	Overlapping functional elements and how these can disguise the integration of sequences with unwanted traits.	Several suggestions are provided of how current biorisk policy might be strengthened. Specific questions include Mechanisms and tools for the better identification of such overlapping elements and their activities, Identifying some type of hierarchy of the impact of various concerning functionalities on DNA repair systems and antiviral host, responses, Developing a better comprehension of their combined function,
Translational insights (2)	Concerns involving siRNAs: it is suggested that The deliberate/accidental integration of short RNAs into a viral genome may engender unrecognized biological activities (e.g., MDH3-siRNAs overlapping with the FCS), Recombination with synthetic RNAs may result in their expression of short RNAs with siRNA capacities.	Whereas the engineered integration of de-facto siRNAs into virus to engender in vivo RNAi has been demonstrated, these have not been described within the framework of natural recombination of RNA viruses with synthetic or natural RNAs. Studies are warranted to unravel the factors enabling recombination with such short RNAs. A key open question concerns the nature of RNAs that could be naturally integrated and expressed as siRNAs. Since viruses acquiring the capacity to silence host genes could have immense implications, it would be important to determine factors that facilitate the acquisition, maintenance, targets, and durability of such processes.
Translational insights (3)	Considerations about SARS-CoV-2’s pathogen–host interaction. Several scenarios are described that provide a rational link of how this sequence homology: Could enable the virus to co-opt the host HR pathway, Might overlap with the action of anticancer agents, such as cisplatin, Drive viral evolution.	Paradoxical features of SARS-CoV-2 relative to the suppression of p53 have recently been described. However, the authors were unable to provide a mechanistic explanation. The multifunctionality conferred by the Ambati-type sequence homology may be key to understand SARS-CoV-2’s impact on DNA damage repair pathways, tumorigenesis, and responses to cancer treatments. It offers a theoretical link between the virus, *MSH3* silencing, and cancer treatment. It raises the question of how such multifunctionalities could be a potent addition for studying the virus-host interplay.
Scenarios that call for more experimental caution	Use of synthetic mRNA or viral vectors that contain short RNAs (or its reverse complement) as part of a therapeutic mRNA, vaccine construct or gene-editing tool could be captured by some CoVs. The resulting chimeric viruses may or may not have some evolutionary benefit. The maintenance and propagation of mutants could be selected for via specific lab conditions. Experiments that deliberately block nuclear trafficking of viral proteins (e.g., ivermectin, importin-αβ blockers) may apply selective pressure that favors mutants with an enhanced NLS/FCS, thereby unintentionally selecting for Ambati et al.-type recombination. Use of DNA-damage-response (DDR) inhibitors or chemotherapeutics that impair DDR in infected cells could prolong viral survival and propagation of recombinant mutants with improved nuclear trafficking capacity, such as via the NLS/FCS insert. Large-scale siRNA-library knockdown screens in virus-infected cells could recombine with the viral genome and get expressed as functional siRNAs targeting host genes. Such chimeric viruses could effectively downregulate critical housekeeping or immune processes and drastically enhance their pathogenicity.	These laboratory context could unintentionally create the conditions for the Ambati et al.-type recombination or deliberately be misused. They do not seem to have been recognized as biorisks before.

## Data Availability

Not applicable. This work is a logical and rational analysis.

## Abbreviations

The following abbreviations are used in this manuscript:

CoV	coronavirus
DDR	DNA damage response
dMMR	a deficiency in DNA mismatch repair
DSB	double strand break
HR	homologous recombination
FCS	furin cleavage site
IAV	influenza A virus
miRNA	micro RNAs
MSH3	the DNA mismatch repair protein, MutS Homolog 3
MMR	DNA mismatch repair
NES	nuclear export signal
NLS	nuclear localization signal
OV	oncolytic virus
RNAi	RNA interference,
siRNA	small interfering RNAs
